# Chromium as a severe anthropogenic geochemical anomaly in river sediments near a tannery outfall: spatial attenuation, source apportionment and ecological risk screening

**DOI:** 10.1007/s10653-026-03457-5

**Published:** 2026-09-03

**Authors:** Rahat Khan, Shuvashish Das Shuvo, Md. Samium Basir, Md. Abu Bakar Siddique, Javed Mallick, Hoang Thi Hang, Md. Harunor Rashid Khan, Saad Aldawood, Dhiman Kumer Roy, Narottam Saha

**Affiliations:** 1 Institute of Nuclear Science and Technology, Bangladesh Atomic Energy Commission (BAEC), Savar, Dhaka, 1349 Bangladesh; 2 Department of Environmental Science, Bangladesh University of Professionals (BUP), Mirpur-12, Cantonment, Dhaka, 1216 Bangladesh; 3 Institute of National Analytical Research and Service (INARS), Bangladesh Council of Scientific and Industrial Research (BCSIR), Dhanmondi, Dhaka, 1205 Bangladesh; 4 Department of Civil Engineering, College of Engineering, King Khalid University, Abha, Kingdom of Saudi Arabia; 5 Physics Discipline, Khulna University, Khulna, 9208 Bangladesh; 6 Department of Physics and Astronomy, College of Science, King Saud University, P.O. BOX 2455, 11451 Riyadh, Saudi Arabia; 7 Department of Geology and Mining, University of Barishal, Barishal, 8254 Bangladesh; 8 Center for Mined Land Rehabilitation, Sustainable Minerals Institute, The University of Queensland, Saint Lucia, Brisbane, QLD 4072 Australia

**Keywords:** Fluvial sediments, Tannery effluents, Chromium, Sediment quality, Source apportionment, Ecological risk

## Abstract

Relocating a polluting industry can transfer contamination to a new receiving water body rather than eliminate it, yet the resulting sediment footprint is rarely resolved beyond surface contamination. This study quantified the spatial and depth-resolved sediment footprint of relocated tannery discharge in the Dhaleshwari River, Bangladesh, and distinguished anthropogenic from geogenic elemental signatures. Sixty sediment samples from 18 sites were analysed for 15 elements by neutron activation analysis. Sites were evaluated individually and within four spatial zones (discharge, opposite riverbank, upstream, and downstream) using non-parametric statistics, sediment-quality indices, and Positive Matrix Factorisation (PMF). Chromium was the dominant anomaly, with concentrations at the discharge point reaching ~260 times the average crustal value (mean: 24,514 ± 16,058 µg/g), declining significantly with distance from the outfall and differing among zones. Depth profiles showed no significant vertical trend across the full sediment cores, but significant near-surface enrichment occurred in the near-field cores, consistent with episodic, source-proximal deposition. Half of all samples exceeded the chromium Effects Range Median threshold. Source apportionment attributed only 15.5% of elemental variance to anthropogenic, chromium-dominated inputs, compared with 65.5% to natural weathering and 19.0% to mixed natural-industrial signatures. Thus, a comparatively small anthropogenic contribution governs the sediment’s principal screening-level ecological concern. This pattern is consistent with particle-reactive Cr being rapidly retained near its release point, producing a sharply confined hotspot rather than basin-wide dispersion. Because risk estimates are based on total Cr rather than speciation or bioavailability, they do not demonstrate biological toxicity. The findings support prioritising targeted interventions at the discharge zone over basin-wide remediation for managing industrial river contamination in comparable settings.

**Supplementary Information:**

The online version contains supplementary material available at https://doi.org/10.1007/s10653-026-03457-5.

## Introduction

Rivers frequently attract industrial development because they provide water, transport corridors, and convenient pathways for waste disposal. That same proximity, however, makes riverine ecosystems highly vulnerable to industrial discharge, a burden that falls disproportionately on rapidly industrialising economies with limited regulatory and treatment capacity (Laoye et al., 2025). Among industrial waste streams, tannery effluents are particularly problematic because they often contain elevated chromium together with saline, organic, and metal-rich process residues that can accumulate in the water column, suspended particulates, and bottom sediments (Azom et al., 2012; Rambabu et al., 2023). Even after treatment, tannery wastewater can retain substantial contaminant loads, and where treatment performance is inconsistent, receiving rivers may become long-term sinks for toxic elements and associated ecological stressors (Sheikh et al., 2007; Upadhyay et al., 2025, 2026; Zvinowanda et al., 2009). Because sediments integrate contaminant inputs over time and archive both depositional and post-depositional processes, they provide a particularly valuable medium for evaluating the persistence, transport, and ecological implications of industrial pollution, and for understanding contaminant redistribution under complex hydrodynamic conditions (Liu et al., 2025a; Zhang et al., 2025a, 2025b).

In Bangladesh, the relocation of the tannery industry from Hazaribag to Hemayetpur, Savar, shifted the pollution burden from one river system to another. The new industrial estate was established with a Central Effluent Treatment Plant (CETP), yet concerns remain regarding treatment efficiency, sludge management, and the continued release of contaminants to the Dhaleshwari River (Rahman et al., 2022a, 2022b, 2022c). Previous studies in the Dhaleshwari system have largely focused on water quality, physicochemical characteristics, or aquatic biota, while sediment-based evidence for contaminant transport, vertical persistence, and source attribution remains comparatively limited (Ahsan et al., 2018; Islam et al., 2012; Real et al., 2017). A previous study in the same river reach examined sedimentary responses to tannery discharge using primordial radionuclides (^226^Ra, ^232^Th, and ^40^ K), but a detailed trace-element assessment capable of resolving the pollution footprint, transport gradients, and source structure of tannery-derived contamination is still lacking (Khan et al., 2023b).

Despite numerous studies on tannery contamination in Bangladesh, important gaps remain in understanding how contaminants disperse from concentrated industrial discharge points, how sediment contamination evolves vertically and laterally with distance from source, and how much of the measured elemental burden can reasonably be attributed to anthropogenic versus geogenic inputs, particularly given the interacting roles of hydrodynamic transport, depositional mixing, and contaminant retention in river sediments (Xu et al., 2026; Zhang et al., 2025b). Earlier investigations of the Dhaleshwari system have not resolved these gaps, largely because they rely on spatially sparse surface-only sampling without source apportionment or ecological screening. The present study addresses these limitations through an integrated design combining structured spatial sampling from the tannery outfall, depth-resolved sediment profiling, Positive Matrix Factorisation (PMF) source apportionment, and multi-index ecological screening. Together, these advances move beyond simple concentration reporting and provide a more management-relevant assessment of contaminant distribution, ecological significance, and source structure in a river system affected by a relocated industrial cluster.

This study, therefore, evaluates sediment contamination in the Dhaleshwari River as a case study of industrial-river management in a rapidly urbanising setting. The specific objectives are to (i) quantify the concentrations and spatial distribution of 15 trace and major elements in surface and depth-stratified core sediments of Dhaleshwari River, (ii) assess contamination status and screening-level ecological risk using geo-environmental enrichment indices, contamination indices, and sediment quality guideline-based ecological risk indices, (iii) identify and apportion contamination sources using PMF and complementary multivariate statistics, and (iv) characterise elemental dispersion patterns as a function of depth and distance from the tannery discharge zone, and to interpret the mechanistic pathways governing sedimentary response to industrial inputs. These contributions provide valuable insights for environmental management and remediation planning.

## Materials and methodology

### Study area

The Dhaleshwari River, a 160-km long meandering distributary of the Brahmaputra-Jamuna-River (BJR) system, originates from the active BJR system in the northeastern tip of the Tangail area and flows southward, merging with the Shitalakshya River near Narayanganj before joining the Meghna River (Khan et al., 2023b). With an average width of 144 m and depth of 37 m, the river is a vital tropical fluvial system receiving substantial sediment and water input from the BJR system. The sediments, primarily derived from the Himalayan region, particularly Darjeeling, exhibit lower concentrations of micas and feldspars but higher concentrations of dark minerals, including zircon, epidote, tourmaline, sillimanite, kyanite, and staurolite (Huizing, 1971). The tropical humid climate of this region is marked by an annual average rainfall, around 220 cm (Khan et al., 2023b).

The lower Dhaleshwari River receives industrial effluents from both the Dhaka Export Processing Zone (DEPZ) in Savar and the relocated Hazaribagh tannery cluster, whose transfer to Savar has intensified the pollution load on the river system. A Central Effluent Treatment Plant (CETP) was established to manage tannery wastewater. However, its treatment capacity, ~ 14,000 m^3^ per day, falls far short of effluent generation rates that can exceed 35,000 m^3^ per day during peak production periods. This structural capacity deficit, compounded by the absence of an effective solid waste management system, means that untreated or partially treated tannery effluents continue to be discharged directly into the river (Tuhin, 2025). Once in the aquatic environment, these contaminants are subject to redistribution through complex riverine transport pathways, contributing to persistent sediment contamination along the Dhaleshwari (Zhang et al., 2024; Zhang et al., 2025a).

### Sample collection and processing

**Fig. 1:**
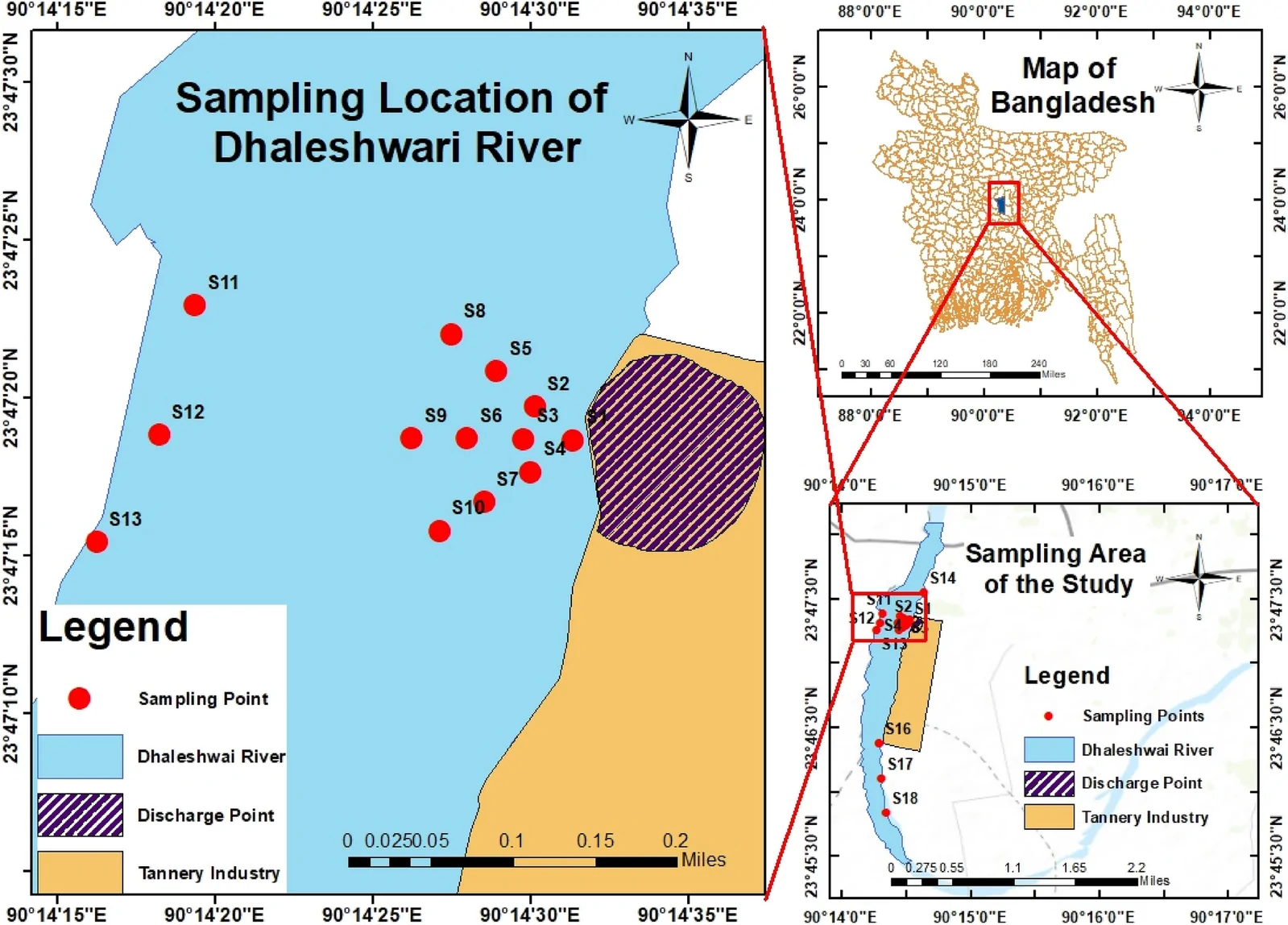
Location of the study area showing sample sites in Dhaleshwari river basin, Bangladesh

Sixty sediment samples from 18 sampling points were systematically collected to examine the spatial dispersion and vertical gradients of the trace elements originating from the tannery effluents in the Dhaleshwari River (Fig. 1 and Table S1). The sampling design comprised three components: (i) transverse dispersion across the river, (ii) lateral dispersion along both banks, and (iii) longitudinal dispersion upstream and downstream of the discharge point. The primary discharge point (S-1), located at the industrial zone, served as the central reference point for all three components.

Transverse samples were collected along a perpendicular transect from S-1 to the opposite bank (S-12), with intermediate sites at 50 m intervals (S-3, S-6, S-9). Lateral dispersion was assessed using two series of sampling points positioned at 45° angles from S-1: left bank sites (S-2, S-5, S-8) and right bank sites (S-4, S-7, S-10), each spaced at 50 m intervals. Two further cross-river samples (S-11, S-13) were positioned at 45° angles on the opposite bank, flanking the transect endpoint (S-12). Longitudinal dispersion analysis was assessed downstream at S-16 (1500 m), S-17 (2000 m), and S-18 (2500 m), and upstream at S-14 (1,500 m) and S-15 (50 m). S-14 was presumed to represent minimal contamination.

For statistical comparison, these eighteen sites were grouped into four spatial zones on the basis of the design above: (i) the discharge zone (S-1 to S-10), comprising the discharge point together with the transverse and lateral transect sites in its immediate vicinity, (ii) the opposite riverbank (S-11 to S-13), comprising the transect endpoint and the two flanking cross-river sites, (iii) the upstream zone (S-14, S-15), and (iv) the downstream zone (S-16 to S-18). These zones are analytical groupings rather than replacements for the individual sampling sites. Zone-level tests evaluate broad spatial contrasts, whereas site identifiers are retained for distance-resolved and depth-resolved analyses.

A ~25-cm sediment core was collected from each of the six key sites spanning the discharge point and the upstream–downstream axis (S-1, S-14, S-15, S-16, S-17, and S-18), following the procedure of Tamim et al. (2016), and sectioned into five 5-cm layers to capture vertical variability. Sites S-2 to S-10 provided shallower 15-cm cores, divided into three 5-cm segments. At the opposite-riverbank sites (S-11, S-12, and S-13), a single composite sample (0–15 cm) was collected at each location without depth sectioning.

The samples were collected using a 100-cm acrylic pipe (diameter: 4 cm) fitted with a rubber eradicator and rubber-cork stopper. All sample handling and pre-treatment adhered to established protocols (Khan et al., 2021a, 2021b; Tamim et al., 2016). Sediment samples were oven-dried at ~70–80 °C to achieve stable mass, then sieved to remove coarse particles, stones, and organic matter. Homogenised sediment samples (550–650 g each) were prepared and ~ 50 mg aliquots were sealed in 1×1 cm high-grade polyethylene envelopes for Instrumental Neutron Activation Analysis (INAA). Repeated measurements (n = 3) of the reference materials (IAEA-RM-Soil-7 and IAEA-RM-SL-1) with comparable matrices were used to guarantee data quality for analytical procedures (Table S2). The detailed methodology can be found elsewhere (Khan et al., 2020; Begum et al., 2021).

### Geo-environmental and ecological indices

This study utilised sediment quality guideline-based indices, geo-environmental indices, and ecological risk indices to assess contamination intensity and screening-level ecological implications (Basir et al., 2024; Khan et al., 2023a, 2023b; Bhuiyan et al., 2015; Kumar et al., 2022). Element-specific indices included the geo-accumulation index (I_geo_) (Müller, 1979), Al-normalised enrichment factor (EF) (Hakanson, 1980), and the contamination factor (CF) (Hakanson, 1980). Site-level indices included the degree of contamination (C_d_) ( Hakanson, 1980), modified degree of contamination (mC_d_) (Abrahim & Parker, 2008), and pollution load index (PLI) (Tomlinson et al., 1980). Indices such as the environmental toxicity quotient (ETQ) (Ali et al., 2018; Maftei et al., 2019), toxic unit analysis (TU) (Islam et al., 2020a, 2020b; Zheng et al., 2008), and mean effects range median quotient (M-ERM-Q) (Islam et al., 2017; Long et al., 2000) were used to assess site-level sediment quality and biological risks. The 60 measured samples were retained as individual observations when reporting sample-level SQG exceedance frequencies. For station-level indices and zone-level comparisons, elemental concentrations were averaged across the available depth intervals at each station to obtain a single site-level value (n = 18). Thus, the site-level risk assessment used depth-averaged concentrations rather than only the uppermost sediment layer.

Upper continental crust values (Rudnick & Gao, 2014) were used as the common reference baseline to facilitate comparison with previous regional studies, while the limitations of a global baseline for site-specific interpretation are acknowledged. Al was selected as the normalising element for EF calculation because it is a widely recognised conservative lithogenic tracer commonly used in sediment contamination assessments. However, Al concentrations are substantially lower at the immediate discharge-point layers (S-1.1–S-1.3: Al = 1.5–1.7%) compared to background sites (median at non-discharge sites: ~7.5%), likely reflecting dilution of the aluminosilicate mineral fraction by tannery-derived waste material. This depletion may inflate EF values specifically at discharge-zone samples, and EF values at S-1 surface layers should therefore be interpreted with caution. The contamination factor (CF) and I_geo_, which do not require element normalisation, are considered the more reliable indices for discharge-zone samples and yield consistent conclusions. Mathematical expressions and classification thresholds are provided in Table S3.

### Positive matrix factorisation (PMF) model

The PMF-model, developed by the USEPA, is a multivariate receptor-model used to quantify source contributions to sediment samples based on source fingerprints (Kim & Hopke, 2007; Men et al., 2018; Yu et al., 2015). It decomposes the contaminant sample data matrix (X) into two matrices: factor contributions (G) and factor profiles (F). This process ensures a chemical mass balance between measured pollutant concentrations and source profiles, as represented in Eq. (1):

1 $${x}_{ij}=\sum_{k=1}^{p}{g}_{ik}{f}_{kj}+{e}_{ij}$$

Here, x_ij_ represents the concentration of the j-th pollutant in the i-th sample; *p* is the number of contributing factors, determined through PCA and repeated PMF runs; g_ik_ is the mass contribution of the k-th factor to the i-th sample; f_kj_ indicates the mass concentration of the j-th element contributed by the k-th factor; and e_ij_ is the residual for the i-th sample and j-th element.

The PMF results are calculated using a weighted least-squares method to minimise the objective function (Q), as shown in Eq. (2), ensuring no sample has significantly negative source contributions:

2 $$Q=\sum_{i=1}^{n}\sum_{j=1}^{m}[\frac{{x}_{ij}-{\sum}_{k=1}^{p}{g}_{ik}{f}_{kj}}{{u}_{ij}}{]}^{2}$$

In Eq. (2), u_ij_ is the measured uncertainty of the j-th pollutant in the i-th sample, related to laboratory or analytical errors. It is quantified using Eq. (3):

3 $${u}_{ij}=\frac{\frac{5}{6}\times MDL}{(\updelta \times {x}_{ij}{)}^{2}+(0.5\times MDL{)}^{2}} \begin{array}{c}{x}_{ij}\le MDL\\ {x}_{ij}>MDL\end{array}$$

Here, δ is the error fraction of the testing method, and MDL is the method detection limit specific to each pollutant (Haque et al., 2022; Men et al., 2020).

### Statistical approaches and data presentation

Elemental concentration data were compiled and screened in SPSS (IBM, version-25), which was used for Pearson correlation analysis and principal component analysis (PCA). Non-parametric spatial and depth-wise tests were performed in R (version 4.5.3), and source apportionment was performed in EPA PMF 5.0 as described in Sect. "Positive matrix factorisation (PMF) model". Distributional properties were assessed before any inferential testing, using Kolmogorov–Smirnov tests with Lilliefors correction and Shapiro–Wilk tests, applied both to raw concentrations and after log10 transformation. Because most variables remained significantly non-normal after transformation, all spatial and vertical inference relied on rank-based methods.

Data were standardised and log_10_-transformed before PCA to reduce scale effects and skewness. PMF was run on measured concentrations and their corresponding analytical uncertainties, without transformation, because the model applies uncertainty-weighted least-squares fitting to the concentration matrix.

For comparison among the four spatial zones, depth-resolved observations were reduced to a single site-level mean to avoid pseudoreplication and were tested using Kruskal–Wallis analysis followed by Bonferroni-adjusted Dunn tests. Distance trends were assessed using Spearman rank correlation. Vertical variation was evaluated only within matched depth structures, because core lengths and the number of sampled intervals differed among stations. Friedman tests were applied separately to the six 25-cm cores and the nine 15-cm near-field cores, and a Wilcoxon signed-rank test compared the shallowest and deepest available intervals across core sites. This approach avoids treating multiple depth intervals from one core as independent replicates.

ArcGIS 10.3 was used solely for cartographic presentation, namely, to prepare the location map, sampling schematic and the elemental distribution map. No statistical analysis was performed in ArcGIS.

## Results and discussion

### Spatial and vertical distribution of trace elements

Major elements (Na, Al, K, Ti) showed variable enrichment relative to crustal references (Table 1). Na concentrations exceeded Upper Continental Crust (UCC) values in most samples except S-14 and S-18, while Al exceeded UCC values in approximately 20% of samples. K surpassed UCC values in 27% of samples and exceeded World Soil Median (WSM) values in 93% of samples. Ti showed higher concentrations than UCC in 55% of samples. Notably, Al and Ti were significantly lower at discharge point (S-1) and at S-15, an upstream-classified site located only 50 m from S-1 (see Sect. "Mechanistic pathway of elemental dispersions and sedimentary response"), than at other locations and geochemical references (e.g., UCC and WSM), likely reflecting near-field dilution by fine-grained tannery waste materials depleted in these lithogenic elements. Several trace elements exceeded UCC values in the majority of samples: Cr (92% of samples), Sb (93%), Co (55%), As (42%), Zn (27%), and Mn (18%). Rare alkali metals (Rb, Cs) and actinides (Th, U) showed approximately 2-fold enrichment relative to both UCC and WSM values (Table 1).

**Table 1:** Concentration of the analyzed chemical elements in sediment samples of the Dhaleshwari River, Bangladesh, along with their characterization in terms of sediment quality guidelines (SQGs) and comparison with those in previous literature

	Na [%]	Al [%]	K [%]	Ti [%]	Cr [µg/g]	Mn [µg/g]	Co [µg/g]	Zn [µg/g]	As [µg/g]	Rb [µg/g]	Sb [µg/g]	Cs [µg/g]	Ba [µg/g]	Th [µg/g]	U [µg/g]
*This work*															
S-1.1	2.3	1.7	0.4	0.2	35,358	330	5.90	251	2.8	31	1.9	1.80	306	4.30	2.1
S-1.2	2.2	1.5	0.4	0.2	37,846	312	7.50	314	3.4	30	1.4	2.00	297	4.20	2.0
S-1.3	2.1	1.6	0.4	0.2	34,102	300	6.70	334	3.9	31	2.1	2.10	271	5.50	2.2
S-1.4	1.6	4.3	1.2	0.4	13,728	556	12.5	219	4.9	75	1.2	4.90	345	11.7	2.1
S-1.5	1.4	7.6	2.4	0.6	1537	783	18.5	93	7.8	153	0.9	9.80	499	19.1	2.5
S-2.1	1.5	7.9	2.2	0.5	888	796	19.9	45	5.5	146	1.7	8.80	461	19.0	3.1
S-2.2	1.2	8.4	2.5	0.7	136	918	23.0	91	7.6	155	1.4	10.9	473	19.5	4.4
S-2.3	1.3	7.3	2.2	0.5	145	765	22.4	101	6.3	160	1.4	10.0	476	17.7	6.0
S-3.1	1.5	8.1	2.2	0.5	1386	867	20.5	124	7.3	154	0.8	9.50	523	20.9	3.1
S-3.2	1.4	8.0	2.2	0.5	346	761	19.7	113	6.5	143	0.8	9.70	452	18.7	2.9
S-3.3	1.3	7.9	2.3	0.6	140.5	842	22.7	96	6.9	158	1.5	10.5	475	18.6	5.3
S-4.1	1.5	4.6	1.3	0.5	8466	536	9.90	136	2.1	66	1.3	3.90	283	16.8	3.0
S-4.2	1.4	8.0	2.2	0.5	346	761	19.7	113	6.5	143	0.8	9.60	452	18.7	2.9
S-4.3	1.6	7.8	2.4	0.5	2159	834	21.9	121	6.4	166	1.2	9.90	549	21.8	3.1
S-5.1	1.9	10.4	2.9	0.6	1855	1075	25.8	151	6.2	187	0.8	11.3	606	27.0	7.4
S-5.2	1.2	6.9	2.1	0.5	400	696	20.7	105	4.9	149	0.9	9.00	466	18.3	3.9
S-5.3	1.2	7.4	2.2	0.6	237	718	21.5	106	7.5	153	1.1	9.00	473	18.8	3.9
S-6.1	1.5	5.4	1.7	0.4	2065	641	7.30	75	3.6	69	0.7	3.70	298	21.7	6.5
S-6.2	1.4	5.4	1.8	0.3	1477	563	12.9	85	2.8	125	1.5	5.50	537	35.7	7.1
S-6.3	1.3	7.3	2.2	0.4	500	827	16.2	92	3.8	126	1.2	7.80	502	20.2	4.1
S-7.1	1.4	6.1	1.8	0.4	1056	636	11.9	95	4.8	95	1.8	5.90	446	30.8	5
S-7.2	1.4	7.6	2.5	0.5	1781	752	13.0	86	4.3	114	1.8	6.90	479	17.6	3.1
S-7.3	1.3	7.3	2.3	0.4	646	747	17.8	116	1.4	147	1.1	8.60	485	17.0	4.3
S-8.1	1.3	6.2	2.0	0.3	543	544	18.6	116	1.3	142	1.0	9.50	575	21.6	4.7
S-8.2	1.4	6.3	2.1	0.3	2286	629	14.6	104	4.9	121	1.5	7.60	451	17.7	3.4
S-8.3	1.3	6.7	1.9	0.4	778	732	14.1	94	4.3	111	1.6	6.80	474	25.5	4.6
S-9.1	1.3	5.0	1.8	0.3	2835	639	6.60	82	5.2	75	0.7	3.60	360	27.2	4.2
S-9.2	1.3	5.5	2.1	0.3	793	461	8.30	53	2.3	95	1.8	4.50	437	10.2	2.5
S-9.3	1.3	5.5	1.9	0.2	1700	628	10.2	73	2.5	93	1.9	5.20	406	12.2	2.2
S-10.1	1.2	5.8	1.9	0.3	2906	499	10.8	91	2.9	109	0.8	6.30	432	50.6	7.4
S-10.2	1.4	5.3	1.9	0.3	1057	491	8.40	70	1.6	96	0.8	4.10	384	17.8	2.8
S-10.3	1.3	7.7	2.5	0.3	149	665	18.6	94	4.1	143	0.6	9.60	507	20.8	4.3
S-11	1.4	8.4	2.7	0.4	305	607	18.7	222	6.8	166	1.3	13.1	643	17.3	5.2
S-12	1.2	9.1	2.7	0.3	183	739	20.8	144	7.4	156	1.2	13.2	568	25.1	4.9
S-13	1.2	8.5	2.3	0.4	97	662	17.5	98	5.4	127	0.7	10.7	452	19.5	4.3
S-14.1	1.3	*7.6*	2.3	*0.4*	*101*	*587*	*16.4*	*95*	*4.1*	144	*2.6*	8.20	449	17.9	3.5
S-14.2	1.2	*7.1*	1.9	*0.4*	*83*	*520*	*11.8*	*82*	*2.5*	121	*3.3*	7.10	403	17.1	3.1
S-14.3	1.3	*7.8*	2.3	*0.4*	*106*	*609*	*14.7*	*97*	*3.3*	126	*2.5*	7.70	476	19.6	3.6
S-14.4	1.3	*7.7*	2.4	*0.5*	*119*	*580*	*15.9*	*114*	*4.1*	143	*0.6*	8.40	487	19.9	3.8
S-14.5	1.3	*7.9*	2.5	*0.4*	*113*	*591*	*17.4*	*109*	*3.6*	138	*1.3*	9.10	499	21.4	4.2
S-15.1	1.4	*5.9*	1.9	*0.3*	*685*	*634*	*7.05*	*121*	*1.2*	93	*2.7*	3.50	379	23.3	2.5
S-15.2	1.3	*5.7*	1.8	*0.2*	*1166*	*339*	*7.36*	*74.2*	*1.6*	91	*3.6*	3.70	341	13.8	2.3
S-15.3	1.3	*5.5*	1.8	*0.2*	*663*	*313*	*6.35*	*137*	*1.7*	85	*2.0*	3.30	329	7.80	2.1
S-15.4	1.2	*5.9*	1.9	*0.3*	*743*	*384*	*7.20*	*77*	*1.1*	103	*1.9*	4.40	383	13.9	1.8
S-15.5	1.1	*5.8*	1.7	*0.3*	*4373*	*391*	*7.60*	*381*	*1.5*	95	*2.4*	4.80	365	8.30	2.1
S-16.1	1.1	*8.5*	2.3	*0.5*	*103*	*566*	*17.9*	*120*	*4.3*	142	*0.7*	9.50	452	16.4	3.5
S-16.2	1.1	*8.7*	2.2	*0.5*	*107*	*496*	*17.2*	*125*	*2.9*	143	*2.9*	10.8	466	15.6	4.6
S-16.3	1.1	*7.8*	2.2	*0.6*	*93*	*588*	*17.8*	*96*	*4.6*	133	*1.4*	8.50	443	14.8	3.3
S-16.4	1.1	*8.4*	2.3	*0.4*	*99*	*661*	*17.8*	*116*	*6.7*	138	*0.6*	9.20	440	14.2	6.1
S-16.5	1.1	*8.3*	2.3	*0.5*	*97*	*727*	*19.4*	*127*	*6.5*	147	*0.7*	9.30	476	13.4	3.4
S-17.1	1.4	*7.0*	2.3	*0.5*	*91*	*503*	*13.7*	*90*	*3.7*	122	*0.5*	7.40	428	17.1	3.7
S-17.2	1.2	*8.6*	2.4	*0.6*	*114*	*544*	*19.3*	*112*	*4.4*	150	*0.6*	11.2	506	16.3	5.1
S-17.3	1.5	*7.3*	2.3	*0.4*	*84*	*529*	*14.3*	*108*	*3.4*	146	*0.7*	7.60	503	23.9	5.3
S-17.4	1.2	*8.8*	2.4	*0.4*	*119*	*595*	*18.7*	*131*	*5.1*	174	*0.9*	11.5	565	18.3	6.1
S-17.5	1.4	*6.9*	2.1	*0.3*	*63*	*497*	*10.1*	*84*	*2.7*	114	*0.5*	0.60	405	16.1	3.9
S-18.1	1.3	*7.9*	2.3	*0.6*	*100*	*584*	*15.4*	*99*	*4.9*	140	*0.7*	8.00	516	23.6	5.6
S-18.2	1.4	*6.9*	2.2	*0.5*	*63*	*466*	*11.4*	*70*	*2.7*	128	*0.7*	7.30	437	19.7	5.5
S-18.3	1.2	*9.1*	2.3	*0.4*	*123*	*616*	*19.45*	*135*	*5.3*	161	*0.7*	11.6	519	18.0	6.3
S-18.4	1.1	*9.2*	2.4	*0.5*	*111*	*570*	*19.3*	*136*	*4.8*	155	*1.1*	12.9	491	17.2	8.9
S-18.5	1.2	*8.6*	2.3	*0.4*	*94*	*589*	*17.2*	*115*	*3.7*	133	*0.5*	9.80	438	17.9	6.1
Mean	1.36	6.96	2.08	0.41	2831	613.14	15.09	120.90	4.27	124.52	1.33	7.69	450.60	18.58	4.11
SD [1σ]	0.24	1.78	0.49	0.12	7918.5	156.70	5.25	62.78	1.86	35.08	0.72	3.06	78.59	7.07	1.59
RSD [%]	17.6	25.6	23.6	29.1	280	25.6	34.8	51.9	43.6	28.2	54.6	39.8	17.4	38.0	38.6
Median	1.31	7.38	2.22	0.42	373	600.59	16.30	105.50	4.20	135.42	1.17	8.28	456.50	18.16	3.85
25th percentile	1.20	5.83	1.90	0.30	108.00	522.25	10.35	91.00	2.80	97.75	0.70	4.98	405.25	16.15	2.93
50th percentile	1.30	7.35	2.20	0.40	373.00	601.00	16.30	105.50	4.20	135.50	1.20	8.30	456.50	18.15	3.90
75th percentile	1.40	8.08	2.30	0.50	1522.00	730.75	19.30	124.75	5.48	148.50	1.78	9.78	499.00	20.88	5.18
Range	1.2	8.9	2.5	.5	37,783	775	19.90	336	6.70	157	3.10	12.60	372	46.40	7.10
Interquartile	.2	2.25	.4	.2	1414	208	8.95	33.75	2.68	50.75	1.08	4.80	93.75	4.73	2.25
Min	1.05	1.54	0.38	0.16	63	300.00	5.85	45.00	1.11	30.00	0.46	0.58	271.00	4.22	1.81
Max	2.27	10.36	2.92	0.65	37,846	1075.00	25.76	381.42	7.81	187.00	3.55	13.22	643.00	50.60	8.90
Errors^a^	1%	2–11%	2–22%	5–12%	0–8%	1–22%	1–5%	2–8%	3–9%	1–7%	3–19%	1–4%	1–7%	0–2%	1–8%
LEL^b^					26	460		120	6						
SEL^b^					110	1100		820	33						
TEL^c^					52.3			124	7.24						
PEL^c^					160			271	41.6						
ERL^d^					81			150	8.2						
ERM^d^					370			410	70						
<LEL					0	11.7		68.3	76.7						
LEL-SEL					25	88.3		31.7	23.3						
>SEL					75	0		0	0						
<TEL					0			73.3	91.7						
TEL-PEL					41.7			21.7	8.3						
>PEL					56.7			5	0						
<ERL					3.3			88.3	100						
ERL-ERM					46.7			11.7	0						
>ERM					50			0	0						
*Literature data*															
Mean (This Work)	1.36	6.96	2.08	0.41	2831	613.14	15.09	120.9	4.27	125	1.33	7.69	450.6	18.6	4.11
UCC^f^	2.43	8.15	2.3	0.4	92	775	17.3	67	4.8	84	0.4	4.9	624	10.5	2.7
World soil median^g^			1.4	0.5	70	1000	8.0	90	6	150	1	4		9.0	2.0
Buriganga river, Bangladesh^h^			2	0.3	788	508	10.5	101	7.1	92		4.8		14.4	4.3
Sela river, Bangladesh^i^					67	634	13.9	67.7	6.76		0.76			21.8	5.36
Teesta river, Bangladesh ^j^				0.35										29	5.36
Poshur river, Bangladesh^k^			2.5	0.48	72.5	649	14.0	69.8	6.73		0.78	10.7		22.6	5.12
Turag River, Bangladesh ^l^	0.98	7.25	1.9	0.40	70.0	768	12.0	780		132	0.61	7.13	415	19.7	3.18
Baram River, Malaysia^m^	0.38	6.34	1.6	0.40	79.1	321	10.8	78.4	6.84	93.3	1.24	6.81	213	10.6	2.73
Yangtze, China^n^				0.67	97.5	853	17.9	125		83.9		8.24		12.1	3.16
Ganga River India^o^					73.9	1263		48.4							
Hindon, India^p^				0.46	49.0	516	8.0	84		103			376	46	6
Gomti River, India^q^					45.5	835		176							
Palar, India^r^				0.39	0.94	358	1.21	0.68		0.6				1.6	2.29
Buyukmelen, Turkey^s^				0.45	170	1040	20.7	64.0	7.11	50.5	0.69				1.3
Kai, Vietnam^t^				0.31	61.5	357	7.65	85.6	4.14	105	1.41	7.5	260	19.5	3.95
Tietê, Brazil^u^				0.41	58.3	668	10.8	332	4.09	64	0.9	3.2		12.8	3.4
Sado, Portugal^v^					61.9		7.59	74.0	7.68	212	0.65	14.6	360	16.7	4.73
Amur, Russia^w^				0.37	36.3	768	6.34	43.38		84.6		4.36	715	6.35	1.74

Italics are collected from Khan et al. (2023b); ^a^Error (analytical uncertainty, in %); ^b^Persaud et al. (1993); ^c^Macdonald et al. (1996); ^d^Long et al. (1995); ^e^ % of sample fall in different category; ^f^Rudnick and Gao (2014); ^g^Bowen (1979); ^h^Tamim et al. (2016); ^i^Islam et al. (2017); ^j^Khan et al. (2020); ^k^Hossain et al. (2023); ^l^Khan et al. (2020); ^m^Prabakaran et al. (2018); ^n^Wu et al. (2013); ^o^Siddiqui and Pandey (2020); ^p^Mondal et al. (2012); ^q^Khan et al. (2020); ^r^Resmi and Achyuthan (2018); ^s^Pehlivan (2010); ^t^Baturin et al. (2014); ^u^Favaro et al. (2018); ^v^Prudêncio et al. (2009); ^w^Sorokina and Zarubina (2011)

Because the elemental dataset and Cr in particular span several orders of magnitude, its distributional properties were characterised before applying inferential statistics. Kolmogorov–Smirnov (with Lilliefors correction) and Shapiro–Wilk tests (n = 60) showed that most variables significantly deviated from normality (*p* < 0.05), both before and after log10 transformation (Table S4-S5). Non-parametric median-based tests further confirmed significant non-random spatial patterns for most elements (Na, K, Ti, Cr, Mn, Co, Zn, As, Rb, Sb, Cs, Th, and U), consistent with mixed anthropogenic–geogenic influences and strong spatial structuring of contamination. Only Al and Ba showed no significant deviation from randomness (*p* > 0.05), suggesting comparatively stable background-controlled distributions (Table S6). Non-parametric and rank-based methods were therefore used throughout this section. Because Cr concentrations at the discharge-point core (S-1) are markedly higher than at all other sites, we additionally tested whether this single core disproportionately influenced the overall statistical and multivariate structure of the dataset. Excluding S-1, Cr remained strongly non-normal (Shapiro–Wilk W = 0.59, *p* < 0.001, n = 55), compared with W = 0.37 (*p* < 0.001) for the full dataset, indicating that the discharge-point core intensifies but is not solely responsible for the dataset’s non-normality. Similarly, excluding S-1 from the PCA reduced the variance explained by the first principal component only moderately (55.4–47.1%), and Cr retained a comparable-magnitude loading on the first two components, indicating that the elemental covariance structure identified by PCA is not an artefact of the S-1 core alone.

Cr shows by far the most extreme spatial variability of all analysed elements, ranging from 63 to 37,846 µg/g across all samples (mean: 2831 ± 7918 µg/g, median: 373 µg/g; Q1–Q3: 108–1522 µg/g, IQR: 1414 µg/g; CV: 280%, n = 60), exceeding UCC (92 µg/g) and WSM (70 µg/g) by more than 30-fold. At the discharge point (S-1), Cr concentrations were exceptionally high (mean: 24,514± 16,058 µg/g, n = 5; range: 1,537–37,846 µg/g), representing ~260-fold enrichment relative to average crustal concentration. The very large gap between the mean and median, together with a CV approaching 280%, confirms that this single site dominates the arithmetic mean and that median and IQR are the more representative summary statistics for Cr in this dataset.

Grouped by the four spatial zones defined in Sect. "Sample collection and processing" (discharge zone, opposite riverbank, upstream, downstream), Cr concentrations varied significantly. The discharge zone, from S-1 to S-10, exhibited much higher mean concentrations than all other zones, with S-1 indicating the maximum level of contamination. The upstream zones S-14 and S-15, the opposite riverbank zones S-11 through S-13, and the downstream zones S-16 through S-18 remained substantially lower. This pattern suggests a geochemical background that is spatially stable with limited tannery influence beyond the immediate discharge area. This spatial gradient was confirmed statistically. Cr concentration was significantly negatively correlated with distance from the discharge point along the upstream–downstream flow axis (Spearman’s ρ = –0.38, *p* = 0.036, n = 30; sites S-1, S-14 to S-18) (Table S7). A non-parametric comparison across the four zones, using site-level mean Cr to treat each site as a single independent unit and avoid pseudo-replication of depth-segment replicates, likewise confirmed significant between-zone differences (Kruskal–Wallis test on site-level mean Cr, χ^2^ = 10.89, df = 3, *p* = 0.01, n = 18 sites), with Dunn’s post-hoc test (Bonferroni-adjusted) confirming significantly higher Cr at the discharge zone than at downstream sites (*p* = 0.024) (Table S7 and Fig. S1), consistent with the spatial attenuation illustrated in the elemental distribution map (Fig. S2).

Vertical Cr distribution also reflected proximity to the discharge point, though less markedly than horizontal pattern. Within-core variability was comparatively low at distal sites but pronounced near the source, with relative standard deviations (RSDs) of roughly 5–24% at the upstream and distal downstream cores compared with roughly 50–117 at the discharge-point core and near-field stations (S-2 to S-10), consistent with uneven contaminant deposition, post-depositional redistribution, and heterogeneous sediment loading close to the source. Because individual depth intervals represent distinct stratigraphic sections rather than strictly replicated observations, depth-wise variation was tested using Friedman’s test, which blocks by site so that only within-core rank order across depth is compared (Table S8). Across the six sites with full 25-cm profiles (S-1, S-14 to S-18), depth had no significant effect on Cr concentration (χ^2^ = 2.53, df = 4, *p* = 0.64)), consistent with the absence of a systematic monotonic increase or decrease with depth at these sites. Within the nine near-field cores (S-2 to S-10), however, a significant depth effect was detected (χ^2^ = 6.89, df = 2, *p* = 0.032). A paired comparison of the shallowest versus deepest available layer across all 15 core sites confirmed significantly higher Cr concentrations at the surface (Wilcoxon signed-rank test, V = 99, *p* = 0.028; median surface = 1,056 µg/g vs. median deepest = 237 µg/g) (Table S8), indicating that surface enrichment, where present, is concentrated in the near-field lateral/transverse cores rather than uniform across the whole study area. Overall, the most prominent pattern remains the pronounced spatial decline in Cr concentrations with increasing distance from the tannery discharge point, indicating that lateral and longitudinal dispersion processes exert stronger control on sediment contamination than vertical stratification within the upper sediment column.

Other potentially toxic elements showed more moderate enrichment patterns. Excluding the discharge point (S-1), Mn, Co, Zn, As, and Sb generally occurred within narrower ranges than Cr, although Zn and Sb showed localised departures from background values. The rare alkali elements (Rb and Cs) and the actinides Th and U were commonly above crustal or world-soil-median benchmarks, but their broader spatial consistency and multivariate associations suggest that they were influenced mainly by sediment provenance and mineralogical controls rather than by tannery-specific enrichment. Overall, the elemental dataset indicates a dominant Cr hotspot superimposed on a more regionally controlled geochemical background.

### Geo-environmental enrichment and pollution assessment

Integrating I_geo_, EF, CF, and site-wise indices (C_d_, mC_d_, PLI) with toxicity/risk indicators (TU, ETQ, M-ERM-Q) yields a consistent spatial pattern. Contamination is strongly element-specific and spatially concentrated at the discharge zone, where Cr dominates every metric. The discharge station (S-1) and immediately adjacent near-field stations form a single high-risk hotspot driven almost entirely by Cr. The opposite riverbank, upstream, and downstream zones remain low to moderate risk and largely reflect geogenic background. Mean I_geo_ and EF values are highest for Cr (I_geo_ up to 7.2 at S-1; mean EF 37.3), with CF and PLI classifications placing Cr in the very high contamination class and the site-wise indices (C_d_, mC_d_, PLI) identifying S-1 as the most contaminated site. The toxicity and risk screening indices TU, ETQ, and M-ERM-Q support the same pattern and are detailed in Sect. "Potential environmental consequences". Other elements show only minor enrichment. Zn, Na, and Sb show modest, localised enrichment consistent with tannery discharge chemistry. Al, K, Ti, Co, Ba, Rb, Cs, Th, and U show low EF and CF values, indicating dominant geogenic control through mineralogical composition, sediment supply, and natural weathering rather than direct industrial loading (Haghnazar et al., 2022a; Khan et al., 2021a).

As detailed in Sect. "Geo-environmental and ecological indices", Al normalisation may be biased at S-1 due to local Al depletion. EF values at the discharge point are therefore interpreted with caution, using CF and I_geo_ as the primary corroborating indices. Elsewhere, where Al depletion is not observed, EF is retained as a complementary line of evidence.

**Fig. 2:**
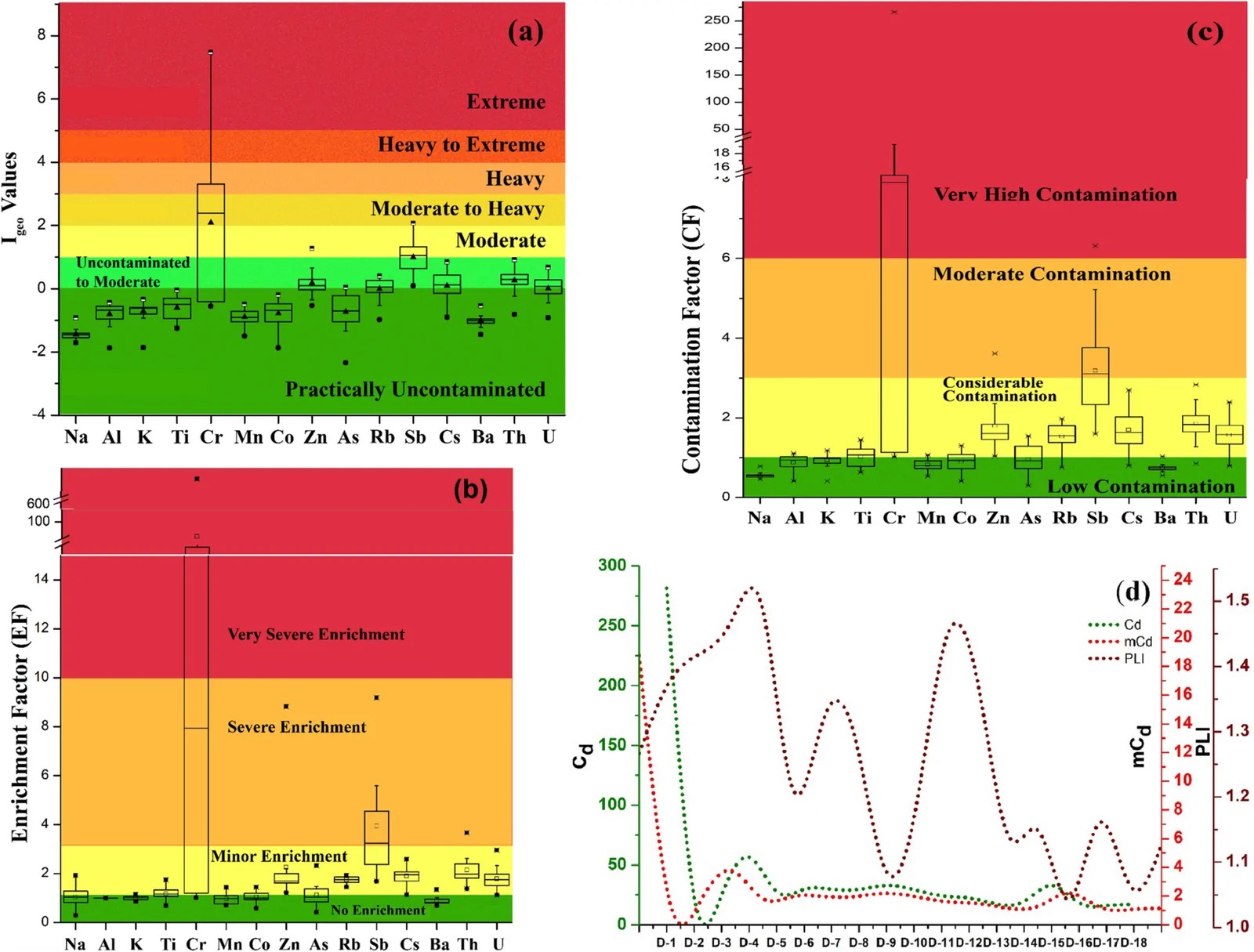
Geo-environmental indices for the sediment samples in the Dhaleshwari River; **a** Geo- accumulation index (I_geo_, mean value of n = 18); **b** Enrichment Factor (EF, mean value of n = 18); **c** Contamination Factor (CF, mean value of n = 18); **d** Values of C_d_, mC_d_, and PLI indices in the riverine sediment sample

These results show strong agreement across indices. Stations identified as high risk by I_geo_ for Cr are also high risk on EF, CF, and the toxicity indices. This consolidated pattern supports a single station level interpretation, with the discharge zone forming the contamination hotspot and the opposite riverbank, upstream, and downstream zones forming the geogenic background. This agreement across indices highlights a sharply delimited Cr-driven contamination hotspot rather than widespread pollution affecting many elements throughout the river reach (Fig. 2).

### Source apportionment by PMF

The PMF analysis was performed using the elemental concentration and their corresponding analytical uncertainties as model inputs. Prior to PMF modelling, Principal Component Analysis (PCA) was conducted, and three principal components with eigenvalues greater than 1 were identified (Fig. S3 and Table S9). These findings supported the selection of a three-factor solution in the EPA PMF 5.0 model for source apportionment and identification of potential pollution sources (Fig. S4 and Table S10) (Anik et al., 2022; Akhi et al., 2024; Basir et al., 2024; Khan et al., 2023b). To assess model robustness and stability, 20 base runs and 20 bootstrap (BS) runs were performed. All base runs converged successfully, indicating satisfactory model stability and reproducibility. The Q(Robust) values ranged from 268.150 to 268.158, while Q(True) values varied only between 273.195 and 273.196, demonstrating minimal variability among repeated runs. Furthermore, the Q(True)/Qexp ratio remained nearly constant (≈0.405), suggesting a stable model fit. The signal-to-noise (S/N) ratios for all analysed elements exceeded 2, indicating that all species were classified as “strong” variables in the PMF model. Additionally, the coefficients of determination (R^2^) between observed and predicted concentrations were greater than 0.75 for all elements, confirming satisfactory model performance and reliable source apportionment results. Scaled residuals were examined and found to be generally centred around zero without systematic bias, supporting the adequacy of the three-factor solution. The consistency of the Cr-dominated anthropogenic factor across all 20 bootstrap iterations provides evidence of robust source identification even in the presence of the high-leverage discharge-point observations at S-1. Rotational ambiguity was not additionally constrained by displacement (DISP) analysis. The stability of the Cr-dominated factor is nevertheless supported by base-run convergence, Q stability, signal-to-noise and scaled-residual diagnostics, together with its recovery across all 20 bootstrap iterations. Because these diagnostics do not substitute for DISP, the potential for unresolved rotational ambiguity remains a limitation of the present PMF analysis.

**Fig. 3:**
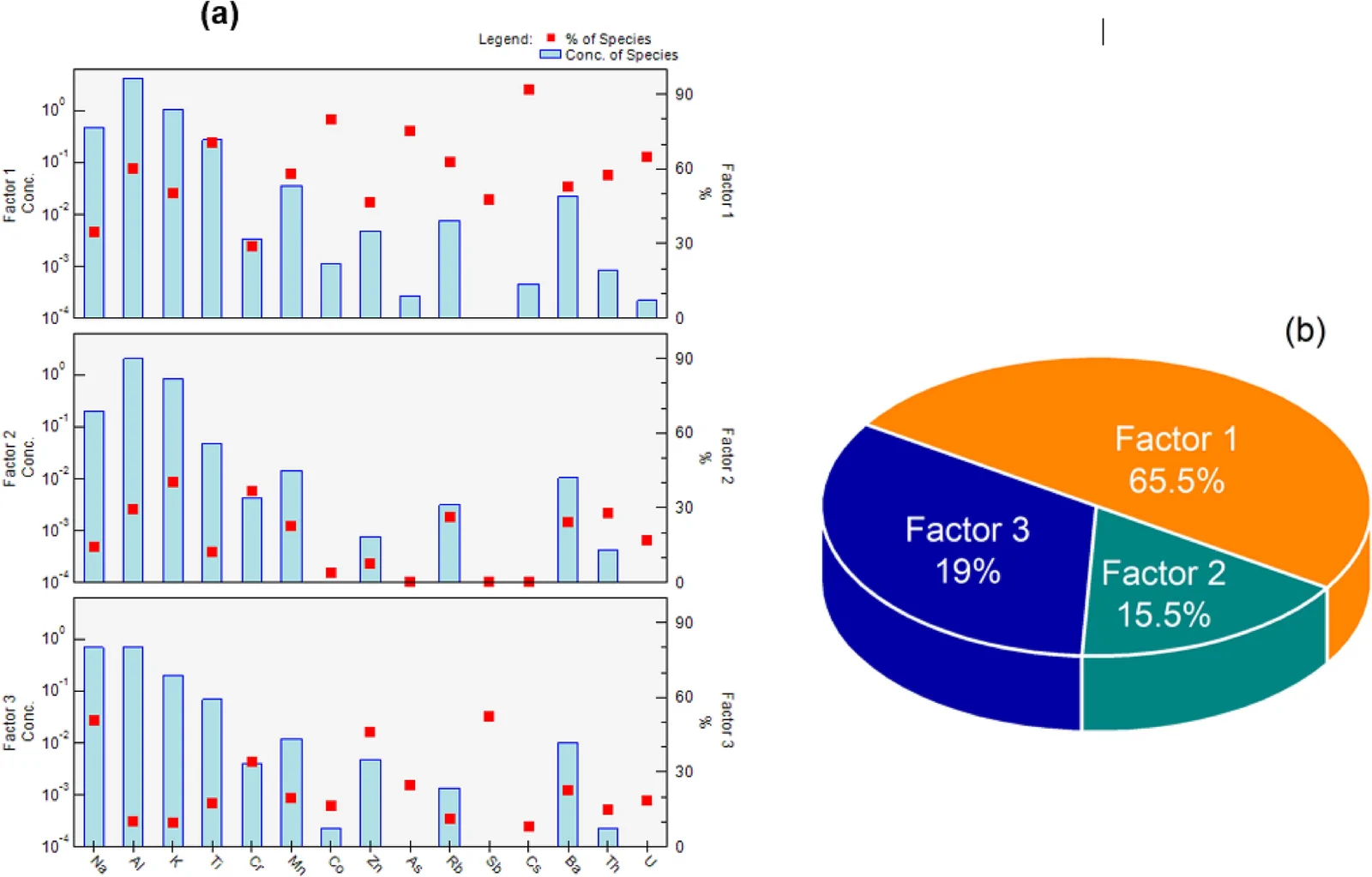
Positive matrix factorization (PMF) results; **a** Contributions of factors to each metal(oid)s and **b** Total contributions of factors to the analyzed metal(oid)s in river sediment

Source 1 had high loading values for Cs (89.27%), Co (85.22%), Ti (81.70%), Rb (78%), Al (75.95%), As (75.89%), U (75.74%), Th (71.04%), and K (66.72%) (Fig. 3a, Table S10). These elements are predominantly derived from the biogeochemical weathering of natural rocks. For instance, Al, a major component derived from the natural erosion of parent rock (Xiao et al., 2019a; Xiao et al., 2019b), exhibited a strong positive correlation with K (r = 0.937; *p* < 0.01) (Table S11). Additionally, both Al and K exhibited mild EF (Fig. 2b) and CF values (Fig. 2c), suggesting their association with clay minerals (aluminosilicate / silicates) (Khan et al., 2019b; Tamim et al., 2016). Significant positive correlations were also observed between Ti and K (Table S11), which, along with low EF and CF values, imply that minerals such as rutile (TiO_2_), ilmenite (FeTiO_3_), and some light/clay minerals like kaolinite (Al_2_O_3_.2SiO_2_.2H_2_O) and illite may have contributed to their origin (Habib et al., 2019, 2022; Habib & Khan, 2021; Khan et al., 2022; Liu et al., 2017; Zhao & Zhen, 2015). Further, Al showed strong positive correlations with Rb (r = 0.921; *p* < 0.01), Cs (r = 0.850; *p* < 0.01), Co (r = 0.792; *p* < 0.01) (Table S11), suggesting the role of natural weathering processes in their accumulation (Khan et al., 2020; Pan et al., 2016; Tamim et al., 2016). Lithophilic elements such as Th and U, known for their low reactivity and insolubility under redox fluvial conditions, exhibit unique behaviour in the oxidising environments. While Th remains largely insoluble as Th^4⁺^, U is more susceptible to differentiation in oxidising conditions, forming soluble oxyanion complexes (Khan et al., 2021a, 2021b; Richards et al., 2020). A strong positive correlation (r = 0.589; *p* < 0.01) between the lithophiles Th and U (Table S11) suggests sedimentary characteristics influenced by minerals such as Th-rich monazite [(LaCeNdTh)PO_4_] and U-rich zircon (ZrSiO_4_) (Khan et al., 2021a, 2022; Ramasamy et al., 2014). The Th/U ratio, a diagnostic indicator of redox conditions, was measured as 4.52 in the Dhaleshwari River benthic sediments, exceeding the Upper Continental Crust (UCC) ratio of 3.89. Ratios below 2 signify an anoxic environment, while ratios above 2 indicates an oxic environment (Adelabu et al., 2021; Khan et al., 2022). Elevated Th/U ratio in this study suggests an oxidising aquatic environment. Under such conditions, Th remains largely unaltered, while U is mobilised through weathering and leaching processes (Begum et al., 2021; Khan et al., 2021a; Rahman et al., 2020). This mobilisation enhances the Th/U ratio, suggesting the prevalence of monazite and other Th-bearing minerals (Khan et al., 2021a).

The partitioning of As within this lithogenic factor rather than within the Cr-dominated anthropogenic factor should be noted, because arsenic-bearing biocides and preservatives are documented co-contaminants of leather processing in other tannery-affected systems (Islam et al., 2017). In the present study, however, As shows no distinct concentration maximum at the discharge point, and its mean concentration across the study reach (4.3 µg/g) remains below the Lowest Effect Level of 6 µg/g (Sect. "Potential Environmental Consequences"). Consistent with this spatial pattern, PMF assigns 75.89% of As to the lithogenic factor together with Al, K, Rb and Cs, while As is not appreciably associated with the Cr–Zn assemblage defining the anthropogenic factor. These observations indicate that no appreciable tannery-associated As contribution is resolved above the lithogenic background in the present dataset. Nevertheless, a minor anthropogenic As contribution below the resolving capability of the three-factor solution, or masked by the regional lithogenic signal, cannot be excluded.

Source 2 was dominated by Cr (46.06%), with additional contributions from Ba (21.53%) and Zn (19.90%) (Fig. 3a; Table S10). This factor is interpreted as the principal anthropogenic source because Cr is the diagnostic contaminant of tannery processing and its highest concentrations occur at the discharge zone. The strong association between Cr and Zn (r = 0.714, *p* < 0.01) suggests co-transport of industrial contaminants, although Zn is also likely influenced by broader sediment interactions and is therefore less source-specific than Cr (Gleyzes et al., 2002; Mohiuddin et al., 2011; Tamim et al., 2016). The strong dominance of chromium in this factor is consistent with studies showing that anthropogenic contaminants often preserve source-specific signatures despite subsequent sediment transport and mixing processes (Xu et al., 2026; Zhang et al., 2025b).

Source 3 was predominantly associated with Sb (55.18%) and Na (37.71%), with smaller contribution from Cr and Zn (Fig. 3a; Table S10). This factor likely represents a mixed source category that combines natural sediment composition with chemically modified material affected by effluent discharge. Sodium is predominantly derived from silicate minerals such as Na-mica and feldspar (Saha & Pal, 2019; Liu et al., 2025b), whereas Cr and Zn are primarily attributed to tannery effluent inputs. The association of Na with effluent-related chemistry is plausible, but because total elemental concentrations alone do not resolve mineralogical hosts or chemical speciation, this factor is interpreted conservatively as mixed rather than uniquely industrial.

Overall, the PMF model attributed 65.5% of the elemental signal to predominantly natural sources, 15.5% to anthropogenic sources, and 19.0% to mixed sources. However, these percentages represent the relative contribution of each source factor to the total elemental composition of the sediments and should not be interpreted as a direct measure of ecological risk or pollution severity (Wu et al., 2026). Therefore, while the sediment matrix remains largely controlled by natural detrital and lithogenic materials, the most ecologically significant contamination signal is concentrated within the Cr-rich anthropogenic and mixed-source components.

### Potential environmental consequences

Consensus-based sediment quality guidelines were used to screen the sediment quality of the Dhaleshwari River (Long et al., 1995; Macdonald et al., 1996; Persaud et al., 1993). These guidelines provide concentration thresholds associated with increasing frequencies of adverse biological effects in sediment toxicity datasets. Elemental concentrations below the ERL (Effects Range Level), TEL (Threshold Effect Level), and LEL (Lowest Effect Level) thresholds suggest infrequent ecotoxicological effects. Conversely, exceeding the ERM (Effects Range Medium), PEL (Probable Effects Level), and SEL (Severe Effect Level) thresholds suggest antagonistic ecotoxicological effects. In this study, the SQG screening focused on Cr, Mn, Zn, and As because these elements have widely used sediment guideline values and are relevant to the observed contamination pattern. (Table 1).

Chromium exceeded all relevant guideline thresholds by a substantial margin (Table 1). Of 60 samples, 15 samples (25%) had Cr concentrations between LEL and SEL, 25 samples (41.7%) fell between TEL and PEL, and 28 samples (46.7%) were within the ERL to ERM range. Notably, half of the 60 analysed samples exceeded the ERM threshold. This magnitude of contamination is consistent with recent reports of severe, effluent-driven Cr enrichment in industrially impacted soils and sediments elsewhere, where enrichment or contamination factors for Cr near point-source industrial discharge have similarly reached tens-fold above background (Elbagory et al., 2026). These findings suggest that Cr contamination in the study area might pose substantial risks to environmental health (Hu et al., 2026; Zhan et al., 2025), indicating a high probability of adverse biological effects under this screening-level SQG framework. However, because this assessment is based on total Cr concentration rather than chromium speciation (Cr(III) versus the more toxic and bioavailable Cr(VI)), sediment binding phase, redox condition, or direct bioavailability measurements, these SQG-based results should be interpreted as screening-level evidence of ecological risk rather than direct proof of biological toxicity. Elevated total Cr has been associated with biological effects such as bacterial DNA damage elsewhere (Khan et al., 2023b; Rahman et al., 2022a), but confirming such effects in this system would require dedicated speciation and toxicity data.

Mn concentrations averaged 613 μg/g, exceeding the LEL (460 μg/g) but remaining below the SEL (1100 μg/g) (Table 1). Approximately 88.3% (n = 53 of 60) of the samples fell within the range of SEL and LEL and none surpassed the SEL threshold. These concentrations are comparable with sediment quality data from global river systems, including the Amur River (Russia, Sorokina & Zarubina, 2011), the Buriganga River (Bangladesh, Tamim et al., 2016), the Buyukmelen River (Turkey, Pehlivan, 2010), and the Tietˆe River (Brazil, Favaro et al., 2018). Consequently, Mn contamination in the middle to lower section of the river may intermittently impact the local ecosystems.

Zn and As concentrations were relatively low compared to Cr and Mn. The mean Zn concentration (120.9 μg/g) was marginally above the LEL (120 μg/g), while the mean As concentration (4.3 μg/g) was below the LEL (6 μg/g) (Table 1). For Zn, 31.7% (n = 19) of samples were between LEL and SEL, while 21.7% (n = 13) were between TEL and PEL, and 11.7% (n = 7) fell between ERL and ERM. For As, 23.3% (n = 14) of samples were between LEL and SEL, and 8.3% (n = 5) were between TEL and PEL. Although these concentrations indicate limited ecotoxicological effects, sediment contamination by As and Zn exceeded the TEL in more than 10% of samples, classifying the area as contaminated (Islam et al., 2017).

**Fig. 4:**
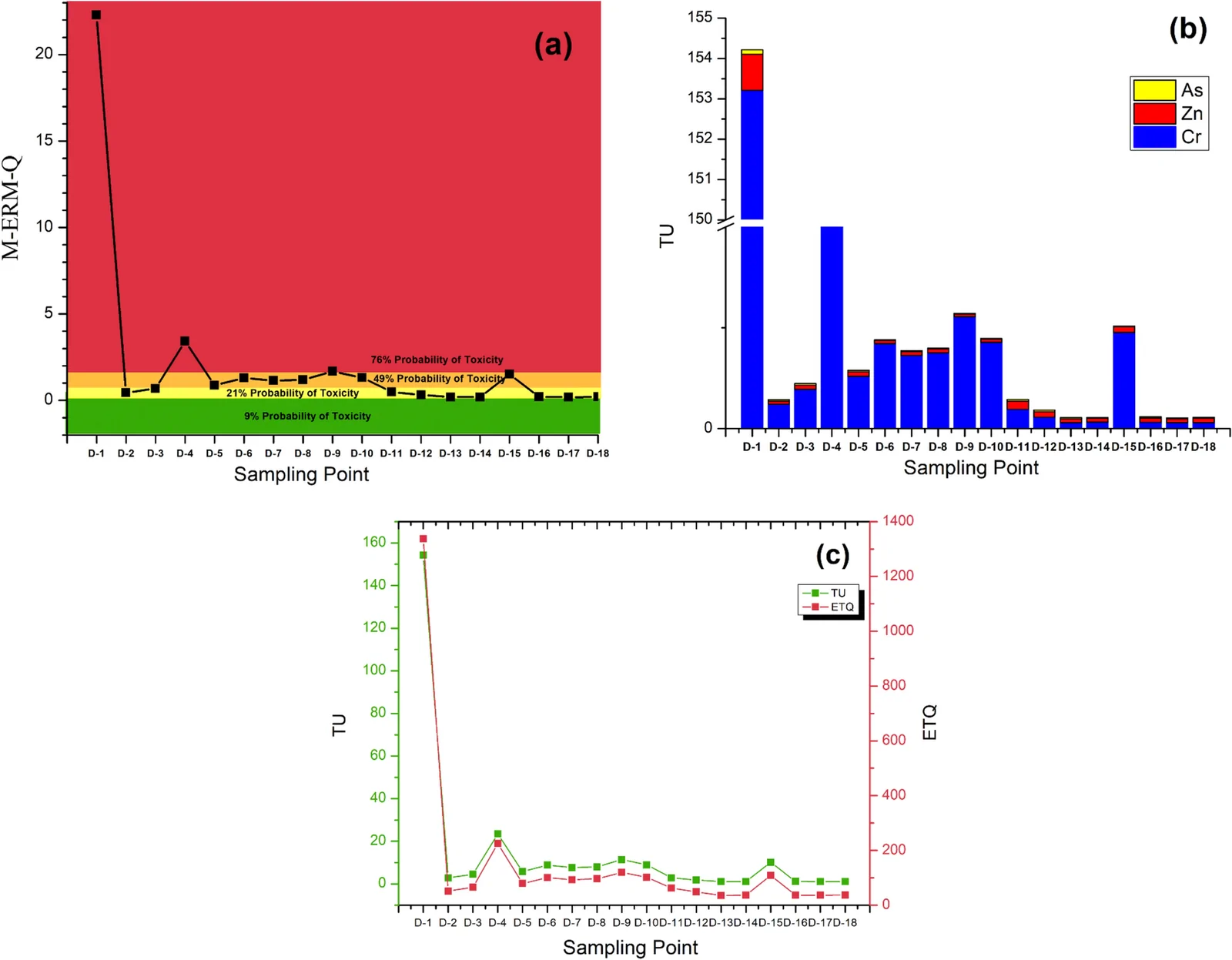
SQGs based indices, ecological indices, and carcinogenic health risks; **a** Mean ERM Quotient (M-ERM-Q); **b** Toxic units (TUs) for As, Zn, and Cr in the analyzed sediments; **c** Sum of toxic units (∑TUs) and environmental toxicity quotient (ETQ) indices in the riverine sediment samples

The sediment quality evaluation was further substantiated by SQG-based indices, including total toxic units (ΣTU) and mean ERM quotients (M-ERM-Q) (Fig. 4) (2020b; Islam et al., 2017; Kumar et al., 2021b; Maftei et al., 2019). These station-level indices were calculated from the depth-averaged concentrations described in Sect. "Geo-environmental and ecological indices" rather than from the uppermost layer alone. ΣTU values ranged from 1.07 to 154.23 (average: 14.23), indicating variable toxicity levels (low, moderate, and high). ΣTU values ranged from 1.07 to 154.23 (mean: 14.23), with the maximum at S-1 and the minimum at S-17 (Fig. 4b). Environmental toxicity quotient (ETQ) values followed the same spatial pattern, with S-1 (1337.72) far exceeding the remaining stations. The next highest ETQs values occurred at S-4 (225.23), S-9 (119.78), and S-15 (108.88), while distal upstream/downstream and opposite-bank sites were generally lower (Fig. 4c). These indices therefore identify the discharge zone, particularly S-1, as the toxicity hotspot rather than indicating uniform toxicity across the river reach (Ali et al., 2018; Maftei et al., 2019Table S3).

Mean ERM-Quotients (M-ERM-Q) ranged from 0.19 (S-13, 14, 17) to 22.3 (S-1), with an average of 2.09 (Fig. 4a). Using the published M-ERM-Q interpretation, 44.4% (n = 8) of sites fell in the 0.11–0.50 class associated with a 21% probability of toxicity, 38.9% (n = 7) fell in the 0.51–1.5 class associated with a 49% probability, and 16.7% (n = 3) exceeded 1.5, corresponding to a 76% probability (Long et al., 2000). These findings suggest the potential biological effects of various toxicants in the Dhaleshwari River sediment (Gao & Chen, 2012; Islam et al., 2017).

### Mechanistic pathway of elemental dispersions and sedimentary response

The spatial patterns observed in the Dhaleshwari River sediments indicate that elemental dispersion from the tannery discharge zone is governed primarily by advective transport in the water column, followed by dilution, sedimentation, and element-specific retention within the bed sediment. Once released with tannery effluent, dissolved and particulate constituents are transported by river flow and progressively redistributed according to local hydrodynamic conditions, including current velocity, turbulence, and depositional setting (Förstner and Salomons, 2011; Tucker and Jones, 2023). The strongest evidence for this process is the statistically significant concentration gradient away from the discharge point established in Sect. "Spatial and vertical distribution of trace elements", particularly for Cr, which shows extreme enrichment at S-1 and a significant decline with distance and with zone.

Among the analysed elements, Cr is the clearest marker of tannery-derived contamination. At the discharge point (S-1), Cr, Zn, Na, and Sb were enriched by 15.9, 2.6, 1.9, and 1.6 times, respectively, relative to the local geological baseline (Table 1). In contrast, mean concentrations at the surrounding sites, including upstream, downstream, and opposite-bank locations, were much lower, although Cr and some associated elements still remained above baseline values. This sharp decline away from S-1 indicates that the discharge point functions as the principal contamination hotspot and that rapid dilution and depositional redistribution occur after effluent enters the river. The especially steep gradient for Cr is consistent with direct input from leather-processing waste and limited dispersion relative to the point source. The data suggest that the dominant pollution footprint is spatially concentrated near the discharge zone, with contamination intensity decreasing downstream as distance from the source increases.

The vertical sediment profiles further support this interpretation. Within-core variability (RSD) is high at the discharge-point core and the near-field stations (roughly 50–117%) and low at the upstream and distal downstream cores (roughly 5–24%), indicating that sediments closer to the source experience more heterogeneous depositional conditions. However, the Friedman test found no significant, systematic depth trend across the six sites with complete 25-cm profiles (S-1, S-14 to S-18), including the discharge point itself, whereas a significant depth effect was detected specifically within the nine near-field cores (S-2 to S-10). This distinction is scientifically informative because at the discharge point and the other long-profile sites, contaminated material appears to be deposited episodically and unevenly, reflecting variable effluent release, short-range settling of suspended solids, and local hydraulic disturbance, without settling into a consistent surface-versus-depth pattern. The near-field lateral and transverse cores, by contrast, show a more consistent directional depth signal, consistent with the significant surface enrichment identified in Sect. "Spatial and vertical distribution of trace elements". The lower variability at distal sites indicates greater mixing, weaker direct source influence, or longer-term depositional averaging. Accordingly, the sedimentary response appears to be controlled not only by distance from the tannery outlet but also by local depositional dynamics that regulate how contaminated fine material is retained within the sediment column, with the specific expression of this control differing between the discharge-point core and the near-field lateral cores (Zhuo et al., 2026; Lei et al., 2026).

Element-specific behaviour also helps explain why the dispersion patterns differ among metals. Chromium shows the strongest and most localised enrichment, whereas Zn, Na, and Sb display more moderate spatial contrasts. This likely reflects differences in their mode of occurrence in the effluent, partitioning between dissolved and particulate phases, and affinity for sedimentary binding phases. Chromium released from tannery processes is commonly associated with particulate and reactive phases that are efficiently scavenged into bed sediments near the discharge point, producing a strong concentration maximum close to source. Zinc may remain more widely distributed because it can associate with multiple sediment fractions, including clays, Fe–Mn oxides, organic matter, and carbonate phases, allowing broader redistribution after discharge. Sodium is more strongly influenced by solution chemistry and dilution, which may explain its comparatively weak and less persistent sedimentary signal away from S-1. Antimony showed a less systematic decline than Cr, suggesting that its behaviour may be influenced by a combination of background sediment composition and variable mobility rather than by direct tannery input alone.

Sediment texture and hydrodynamic sorting may also contribute to the observed patterns. Fine-grained particles generally have higher surface area and stronger capacity to retain trace elements than coarse grains. Under turbulent fluvial conditions, these finer contaminated particles can remain in suspension longer and be transported farther before deposition, whereas coarser particles settle more rapidly (Habib et al., 2022; McLennan et al., 1993; Zhi et al., 2025). This process provides a plausible explanation for why contamination remains detectable beyond the discharge point, even though the strongest enrichment is concentrated at S-1. It also helps explain why some elements exhibit broader dispersion than Cr, particularly where they are preferentially associated with fine sediment fractions.

The relatively uniform concentrations of Th and U across most sites, except near the discharge zone, indicate that these elements are controlled predominantly by natural sediment provenance rather than tannery discharge. Their limited spatial variability across upstream, downstream, and opposite riverbank zones supports a regional fluvial source with local industrial overprinting. As noted in Sect. "Spatial and vertical distribution of trace elements", S-15 is classified as upstream but lies only 50 m from S-1 and therefore retains near-field characteristics and shows comparatively elevated ETQ (Sect. "Potential Environmental Consequences"). The low mean U/Th ratio (0.21) is also consistent with oxic depositional conditions, under which U is relatively more mobile than Th (McLennan et al., 1993; Ramasamy et al., 2014). This interpretation aligns with the PMF results, which show that most elements are predominantly associated with natural lithogenic sources, while Cr is strongly associated with the anthropogenic source factor.

Overall, the sedimentary response in the Dhaleshwari River is consistent with a strong point-source input superimposed on a largely natural fluvial sediment background. The tannery outlet produces an intense but spatially decaying contamination signal, with Cr as the dominant anthropogenic tracer. Downstream dilution, hydrodynamic sorting, and depositional mixing progressively reduce this signal, while naturally sourced lithogenic elements remain comparatively stable across the river system. This mechanistic interpretation is supported by the concentration gradients, geo-environmental indices, and source-apportionment results, emphasizing that controlling contaminant release at the discharge point is essential for reducing sediment contamination in the receiving river. It also highlights the importance of integrating sediment geochemistry with process-based transport modeling and predictive environmental assessment approaches (Dai et al., 2026; Lyu et al., 2026; Zhi et al., 2026).

## Conclusions and future research

River sediments adjacent to the relocated Hemayetpur tannery estate show a chromium-dominated contamination hotspot centred on the discharge zone rather than broadly distributed through the river system. Among the fifteen elements measured, Cr dominated the concentration anomaly, enrichment and contamination classifications, and screening-level ecological-risk metrices. Mean discharge-point Cr reached approximately 260-fold above the average crustal concentration, and half of all samples exceeded the Cr Effects Range Median threshold. Spatial and depth-resolved statistical tests, geo-environmental indices (I_geo_, EF, CF), and sediment quality guideline-based risk indices consistently identified the discharge zone as the principal area of concern relative to the opposite riverbank, upstream, and downstream zones.

Source apportionment by PMF attributed 65.5% of the total elemental variance to natural weathering, 15.5% to a Cr-dominated anthropogenic factor, and 19.0% to a mixed sources. These percentages describe source contributions to bulk elemental composition rather than ecological risk. The comparatively small anthropogenic fraction nevertheless contains the dominant Cr anomaly and therefore governs the principal screening-level ecological concern. The depth-resolved profiles further showed that significant near-surface Cr enrichment was restricted to the near-field cores rather than expressed uniformly across the wider river reach, reinforcing lateral proximity to the discharge point as the principal control on the observed Cr distribution.

These findings carry direct management implications. Without intervention, continued Cr loading is likely to further degrade sediment quality at the discharge zone. This could have downstream consequences for aquatic biota and, by extension, for communities that rely on the river for fisheries and other ecosystem services. More broadly, the results show that centralised effluent treatment alone does not guarantee protection against localised, high-intensity contamination. Rigorous enforcement of treatment performance and compliance is equally important. This is a direct concern for industrial relocation programmes in Bangladesh and other developing economies with comparable regulatory capacity.

This assessment is based on total elemental concentrations in sediment, and its conclusions should be read within that scope. It does not, on its own, establish bioavailability or biological toxicity. These depend on chromium speciation, specifically Cr(III) versus the more mobile and toxic Cr(VI), together with sediment binding phase, redox condition, and organic matter content, none of which were measured here.

Confirming the ecological consequences of this contamination will require four complementary lines of future work. First, chromium speciation and sediment binding-phase analysis are needed to establish the proportion of total Cr that is bioavailable and to determine actual, rather than screening-level, ecotoxicological risk. Second, direct toxicity testing through sediment bioassays and benthic community surveys would test whether the elevated risk indices identified here (ΣTU, M-ERM-Q) correspond to measurable biological effects. Third, seasonal and hydrological monitoring is needed to determine whether the localised contamination pattern documented here persists, expands, or attenuates under varying discharge, flow, and depositional conditions, and whether Cr bioaccumulates in locally consumed fish. Fourth, complementary analysis of additional trace elements such as Fe and Ni, together with XRD-based mineralogical characterisation, would refine the natural detrital background resolved by PMF and strengthen confidence in the anthropogenic source attribution. Together, these steps would extend the present spatial and source-apportionment framework into a fully management-relevant risk assessment for tannery-impacted river systems.

## Supplementary Information

Below is the link to the electronic supplementary material.

Supplementary file1 (DOCX 1241 kb)

## Data Availability

All data supporting the findings of this study are available within the paper and its Supplementary Information.

## References

[CR1] Abrahim, G., & Parker, R. (2008). Assessment of heavy metal enrichment factors and the degree of contamination in marine sediments from Tamaki Estuary, Auckland, New Zealand. *Environmental Monitoring and Assessment,**136*(1–3), 227–238. 17370131 10.1007/s10661-007-9678-2

[CR2] Adelabu, I. O., Opeloye, S. A., Oluwajana, O. A., & Ogbahon, O. A. (2021). Paleoredox conditions of paleocene to miocene rocks of eastern Benin basin, Nigeria: implications for chemostratigraphy. *International Journal of Geography and Geology,**10*(1), 24–35. 10.18488/journal.10.2021.101.24.35

[CR4] Ahsan, M. A., Siddique, M. A. B., Munni, M. A., Akbor, M. A., Akter, S., & Mia, M. Y. (2018). Analysis of Physicochemical Parameters, Anions and Major Heavy Metals of the Dhaleshwari River Water, Tangail. *Bangladesh. American Journal of Environmental Protection,**7*(2), 29–39.

[CR5] Akhi, S. Z., Khan, R., Basir, M. S., Habib, M. A., Islam, M. A., Naher, K., Idris, A. M., Khan, M. H. R., Aldawood, S., & Roy, D. K. (2024). Exploring the alteration of environmental radioactivity in terms of compositional elements of heavy minerals in an anthropogenically affected urban river: Radiological and ecological risks assessment. *Marine Pollution Bulletin,**206*, Article 116694. 10.1016/j.marpolbul.2024.116694 39002213

[CR9] Ali, M. M., Ali, M. L., Islam, M. S., & Rahman, M. Z. (2018). Assessment of toxic metals in water and sediment of Pasur River in Bangladesh. *Water Science and Technology,**77*, 1418–1430. 10.2166/wst.2018.016 29528329

[CR10] Anik, A. H., Khan, R., Hossain, S., Siddique, M. A. B., Tamim, U., Islam, A. T., Islam, A. R. M. T., Idris, A. M., & Tareq, S. M. (2022). Reconciling the geogenic and non-crustal origins of elements in an Indo-Bangla transboundary river, Atrai: Pollution status, sediment quality, and preliminary risk assessment. *Environmental Research,**214*, Article 114134. 35998696 10.1016/j.envres.2022.114134

[CR11] Azom, M. R., Mahmud, K., Yahya, S. M., Sontu, A., & Himon, S. B. (2012). Environment impact assessment of tanneries: A case study of Hazaribug in Bangladesh. *International Journal of Environmental Science and Development,**3*, 152–156.

[CR12] Basir, M. S., Khan, R., Akhi, S. Z., Ullah, A. K. M. A., Islam, M. A., Naher, K., Idris, A. M., Khan, M. H. R., Aldawood, S., & Saha, N. (2024). Source specific sedimentary response towards the differential anthropogenic impacts in terms of potentially toxic elements in an urban river. *Marine Pollution Bulletin,**203*, Article 116425. 10.1016/j.marpolbul.2024.116425 38705004

[CR108] Baturin, G. N., Lobus, N. V., Peresypkin, V. I., & Komov, V. T. (2014) Geochemistry of channel drifts of the Kai River (Vietnam) and sediments of its mouth zone. *Oceanology, 54*(6), 788–797. 10.1134/S0001437014050026

[CR14] Begum, M., Khan, R., Roy, D. K., Habib, M. A., Rashid, M. B., Naher, K., Islam, M. A., Tamim, U., Das, S. C., Mamun, S. M. M. A., & Hossain, S. M. (2021). Geochemical characterization of Miocene core sediments from Shahbazpur gas-wells (Bangladesh) in terms of elemental abundances by instrumental neutron activation analysis. *Journal of Radioanalytical and Nuclear Chemistry*. 10.1007/s10967-021-07770-4

[CR15] Bhuiyan, M. A. H., Dampare, S. B., Islam, M. A., & Suzuki, S. (2015). Source apportionment and pollution evaluation of heavy metals in water and sediments of Buriganga River, Bangladesh, using multivariate analysis and pollution evaluation indices. *Environmental Monitoring and Assessment,**187*, Article 4075. 10.1007/s10661-014-4075-0 25416128

[CR16] Bowen, H. J. M. (1979). *Environmental chemistry of the elements*. Academic Press.

[CR18] Dai, H., Zhang, Y., Deng, Y., Guadagnini, A., Yang, J., Pan, Z., Fang, W., Zhang, J., Ye, M., Yuan, S., & Wang, Y. (2026). Gene-informed modeling of denitrification process in groundwater through dynamic flux balance analysis and deep learning. *Water Resources Research,**62*(5), Article e2026WR043411. 10.1029/2026WR043411

[CR20] Elbagory, M., El-Zahaby, H. M., EL-Shafeey, E. S. E., EL-Khateeb, N., El-Dein Omara, A., El-Nahrawy, S., EL-Shafeey, E.L.-S., Zayed, A., Kumar, P., & Singh, J. (2026). Heavy metal contamination in soil and Indian mustard (*Brassica juncea* L. Czern.) irrigated with borewell water and paper mill effluent. *Scientific Reports*. 10.1038/s41598-026-62409-2

[CR22] Favaro, D. I. T., Rocha, F. R., Angelini, M., Henriques, H. R. A., Soares, J. S., Silva, P. S. C., & Oliveira, S. M. B. (2018). Metal and trace elements assessments of bottom sediments from medium Tietˆe River basin, Sao Paulo State, Brazil: Part II. *Journal of Radioanalytical and Nuclear Chemistry,**316*, 805–818.

[CR23] Gao, X., & Chen, C. A. (2012). Heavy metal pollution status in surface sediments of the coastal Bohai Bay. *Water Research,**46*, 1901–1911. 10.1016/j.watres.2012.01.007 22285040

[CR26] Gleyzes, C., Tellier, S., & Astruc, M. (2002). Fractionation studies of trace elements in contaminated soils and sediments: A review of sequential extraction procedures. *TrAC Trends in Analytical Chemistry,**21*(6–7), 451–467.

[CR28] Habib, M. A., Basuki, T., Miyashita, S., Bekelesi, W., Nakashima, S., Phoungthong, K., Khan, R., Rashid, M. B., Islam, A. R. M. T., & Techato, K. (2019). Distribution of naturally occurring radionuclides in soil around a coal-based power plant and their potential radiological risk assessment. *Radiochimica Acta,**107*(3), 243–259. 10.1515/ract-2018-3044

[CR29] Habib, M. A., & Khan, R. (2021). Environmental Impacts of Coal-Mining and Coal-Fired Power-Plant Activities in a Developing Country with Global Context. Spatial Modeling and Assessment of Environmental Contaminants, Chapter 24. Environmental challenges and solutions, Springer Nature Switzerland AG. 10.1007/978-3-030-63422-3_24

[CR30] Habib, M. A., Khan, R., & Phoungthong, K. (2022). Evaluation of environmental radioactivity in soils around a coal burning power plant and a coal mining area in Barapukuria, Bangladesh: radiological risks assessment. *Chemical Geology*. 10.1016/j.chemgeo.2022.120865

[CR31] Haghnazar, H., Cunningham, J. A., Kumar, V., Aghayani, E., & Mehraein, M. (2022a). COVID-19 and urban rivers: Effects of lockdown period on surface water pollution and quality- A case study of the Zarjoub River, north of Iran. *Environmental Science and Pollution Research,**29*, 27382–27398. 10.1007/s11356-021-18286-5 34981401 PMC8723709

[CR34] Hakanson, L. (1980). An ecological risk index for aquatic pollution control- A sedimentological approach. *Water Research,**14*(8), 975–1001.

[CR36] Haque, M. M., Sultana, S., Niloy, N. M., Quraishi, S. B., & Tareq, S. M. (2022). Source apportionment, ecological, and human health risks of toxic metals in road dust of densely populated capital and connected major highway of Bangladesh. *Environmental Science and Pollution Research*. 10.1007/s11356-021-18458-3 35034304

[CR37] Hossain, S., Khan, R., Anik, A. H., Siddique, M. A. B., Tamim, U., Islam, A. R. M. T., Idris, A. M., & Khaleque, M. A. (2023). Natural and anthropogenic contributions to the elemental compositions and subsequent ecological consequences of a transboundary river’s sediments (Punarbhaba, Bangladesh). *Environmental Research,**216*, Article 114444. 36179881 10.1016/j.envres.2022.114444

[CR115] Hu, F., Yang, H., Zhou, X., Zhao, S., Qiu, L., Wei, S., Wang, X., Hu, J., Chen, Y., Hu, H., & Zhou, H. (2026) Pollution transfer and environmental health implications: Network evolution and proximity mechanisms in the Yangtze River Delta, China. *Frontiers in Public Health, 14*, 1770901. 10.3389/fpubh.2026.1770901 PMC12950677 41778135

[CR39] Huizing, H. G. J. (1971). A reconnaissance study of the mineralogy of sand fractions from East Pakistan sediments and soils. *Geoderma,**6*(2), 109–133. 10.1016/0016-7061(71)90029-2

[CR40] Islam, A. R. M. T., Hasanuzzaman, M., Islam, H. M. T., Mia, M. U., Khan, R., Habib, M. A., Rahman, M. M., Siddique, M. A. B., Moniruzzaman, M., & Rashid, M. B. (2020a). Quantifying source apportionment, co‐occurrence, and ecotoxicological risk of metals from upstream, lower midstream, and downstream river segments, Bangladesh. *Environmental Toxicology and Chemistry,**0*, pp-1-14. 10.1002/etc.4814 32633828

[CR41] Islam, A. R. M. T., Hasanuzzaman, M., Islam, H. M. T., Mia, M. U., Khan, R., Habib, M. A., Rahman, M. M., Siddique, M. A. B., Moniruzzaman, M., & Rashid, M. B. (2020b). Quantifying source apportionment, co-occurrence and ecotoxicological risk of metals from up-mid-downstream river segments, Bangladesh. *Environmental Toxicology and Chemistry.,**1*, 1. 10.1002/etc.4814 32633828

[CR42] Islam, M. A., Al-mamun, A., Hossain, F., Quraishi, S. B., Naher, K., Khan, R., Das, S., Tamim, U., Hossain, S. M., & Nahid, F. (2017). Contamination and ecological risk assessment of trace elements in sediments of the rivers of Sundarban mangrove forest, Bangladesh. *Marine Pollution Bulletin,**124*, 356–366. 10.1016/j.marpolbul.2017.07.059 28760588

[CR43] Islam, M. S., Meghla, N. T., Suravi, M. S., & Islam, M. (2012). Status of water quality in the Dhaleshwari River and its effect on aquatic organism. *Journal of Environmental Science and Water Resources,**1*(8), 192–201.

[CR44] Khan, R., Siddique, M. A. B., Chowdhury, Y. F., Ahmed, M. N., Ullah, A. K. M. A., Khan, M. H. R., Islam, A. R. M. T., Habib, M. A., Khan, A. H. A. N., Aldawood, S., & Idris, A. M. (2023a). Fluvial responses towards the tannery effluent: Tracing the anthropogenic foot-prints. *Environmental Pollution*. 10.1016/j.envpol.2023.122673 37793543

[CR45] Khan, R., Hossain, S., Anik, A. H., Phoungthong, K., Islam, A. R. M. T., Saha, N., Idris, A. M., & Alam, M. (2023b). Indexical and statistical approaches to investigate the integrated origins of elements in the sediment of Teesta River, Bangladesh: Sediment quality and ecological risk assessment. *Environmental Science: Processes and Impacts,**25*(4), 832–849. 36897614 10.1039/d2em00475e

[CR46] Khan, R., Islam, H. M. T., & Islam, A. R. M. T. (2021a). Mechanism of elevated radioactivity in Teesta river basin from Bangladesh: Radiochemical characterization, provenance and associated hazards. *Chemosphere,**264*, Article 128459. 10.1016/j.chemosphere.2020.128459 33032211

[CR47] Khan, R., Mohanty, S., & Sengupta, D. (2021b). Studying the elemental distribution in the core sediments of Podampata, Eastern coast of Odisha, India: Potentiality of rare earth elements and Th exploration. *Arabian J. Geosci.,**14*, 81. 10.1007/s12517-020-06371-x

[CR48] Khan, R., Islam, M. S., Tareq, A. R. M., Naher, K., Islam, A. R. M. T., Habib, M. A., Siddique, M. A. B., Islam, M. A., Das, S., Rashid, M. B., Ullah, A. K. M. A., Miah, M. M. H., Masrura, S. U., Bodrud-Doza, M., Sarker, M. R., & Badruzzaman, A. B. M. (2020). Distribution, sources and ecological risk of trace elements and polycyclic aromatic hydrocarbons in sediments from a polluted urban river in central Bangladesh. *Environmental Nanotechnology, Monitoring and Management,**14*, Article 100318. 10.1016/j.enmm.2020.100318

[CR111] Khan, R., Islam, H. M. T., Apon, M. A. S., et al. (2022). Environmental geochemistry of higher radioactivity in a transboundary Himalayan river sediment (Brahmaputra, Bangladesh): Potential radiation exposure and health risks. *Environmental Science and Pollution Research, 29*, 57357–57375. 10.1007/s11356-022-19735-5 35349070

[CR50] Khan, Z., Quasim, M. A., Amir, M., & Ahmad, A. H. M. (2019). Provenance, tectonic setting, and source area weathering of Middle Jurassic siliciclastic rocks of Chari Formation, Jumara Dome, Kachchh Basin, Western India: Sedimentological, mineralogical, and geochemical constraints. *Geological Journal,**55*(5), 3537–3558. 10.1002/gj.3612

[CR51] Kim, E., & Hopke, P. K. (2007). Comparison between sample-species specific uncertainties and estimated uncertainties for the source apportionment of the speciation trends network data. *Atmospheric Environment,**41*, 567–575. 10.1016/j.atmos.env.2006.08.023

[CR53] Kumar, S., Islam, A. R. M. T., Hasanuzzaman, M., Salam, R., Islam, M. S., Khan, R., Rahman, M. S., Pal, S. C., Ali, M. M., Idris, A. M., Gustave, W., & Elbeltagi, A. (2022). Potentially toxic elemental contamination in Wainivesi River, Fiji impacted by gold mining activities using chemometric tools and SOM analysis. *Environmental Science and Pollution Research*. 10.1007/s11356-022-18734-w 35088286

[CR55] Kumar, S., Islam, A. R. M. T., Islam, H. M. T., Hasanuzzaman, M., Ongoma, V., Khan, R., & Mallick, J. (2021b). Water resources pollution associated with risks of heavy metals from Vatukoula Goldmine region, Fiji. *Journal of Environmental Management,**293*, Article 112868. 10.1016/j.jenvman.2021.112868 34089960

[CR56] Laoye, B., Olagbemide, P., Ogunnusi, T., & Akpor, O. (2025). Heavy metal contamination: Sources, health impacts, and sustainable mitigation strategies with insights from Nigerian case studies. *F1000Research,**14*, 134. 40822873 10.12688/f1000research.160148.4 PMC12355173

[CR123] Lei, H., Bao, N., Ben-Dor, E., Peng, S., & Zhao, N. (2026). Soil bidirectional reflectance characteristics and applications in quantitative soil property retrieval: A systematic review. *CATENA, 273*, 110456. 10.1016/j.catena.2026.110456

[CR58] Liu, C., Shan, Y., Li, F., Liu, X., & Nepf, H. (2025a). Submerged flexible canopies produce weaker shear‐layer turbulence and mitigate sediment transport, compared to rigid model canopies. *Geophysical Research Letters*. 10.1029/2025GL117007

[CR117] Liu, M., Qiao, W., Cheng, X., Lv, R., & Meng, X. (2025b). Effect of depositional environment differences on micro-macro rheological behavior of sedimentary soft rocks. *International Journal of Mining Science and Technology, 35*(12), 2179–2198. 10.1016/j.ijmst.2025.10.006

[CR59] Liu, C., Zhang, L., Fan, C., Xu, F., Chen, K., & Gu, X. (2017). Temporal occurrence and sources of persistent organic pollutants in suspended particulate matter from the most heavily polluted river mouth of Lake Chaohu, China. *Chemosphere,**174*, 39–45. 10.1016/j.chemosphere.2017.01.082 28157607

[CR60] Long, E. R., Macdonald, D. D., Smith, S. L., & Calder, F. D. (1995). Incidence of adverse biological effects within ranges of chemical concentrations in marine and estuarine sediments. *Environmental Management,**19*, 81–97.

[CR61] Long, E. R., MacDonald, D. D., Severn, C. G., & Hong, B. C. (2000). Classifying probabilities of acute toxicity in marine sediments with empirically derived sediment quality guidelines. *Environmental Toxicology,**19*, 2598–2601.

[CR62] Lyu, L., Jacinthe, P., Lin, N., Lyu, D., Liu, G., Li, S., Jacinthe, P.-A., Fang, C., Tao, H., Yu, H., Li, Z., & Song, K. (2026). Global inland waters trophic status from space observation: Scientific advances and future challenges. *Water Research,**293*, Article 125419. 10.1016/j.watres.2026.125419 41576817

[CR64] Macdonald, D. D., Carr, R. S., Calder, F. D., Long, E. R., & Ingersoll, C. G. (1996). Development and evaluation of sediment quality guidelines for Florida coastal waters. *Ecotoxicology,**5*, 253–278. 24193815 10.1007/BF00118995

[CR65] Maftei, A. E., Buzatu, A., Buzgar, N., & Apopei, A. I. (2019). Spatial distribution of minor elements in the Tazlău River sediments: Source identification and evaluation of ecological risk. *International Journal of Environmental Research and Public Health,**16*, Article 4664. 10.3390/ijerph16234664 31771109 PMC6926686

[CR106] McLennan, S. M., Hemming, S., McDaniel, D. K., & Hanson, G. N. (1993). *Geochemical approaches to sedimentation, provenance, and tectonics*.

[CR67] Men, C., Liu, R., & Xu, F. (2018). Pollution characteristics, risk assessment, and source apportionment of heavy metals in road dust in Beijing, China. *Science of the Total Environment,**612*, 138–147. 10.1016/j.scito.tenv.2017.08.123 28850834

[CR68] Men, C., Liu, R., & Xu, L. (2020). Source-specific ecological risk analysis and critical source identification of heavy metals in road dust in Beijing, China. *Journal of Hazardous Materials*. 10.1016/j.jhazmat.2019.121763 31818668

[CR70] Mohiuddin, K. M., Ogawa, Y. Z. H. M., Zakir, H. M., Otomo, K., & Shikazono, N. (2011). Heavy metals contamination in water and sediments of an urban river in a developing country. *International Journal of Environmental Science and Technology,**8*(4), 723–736.

[CR107] Mondal, M. E. A., Wani, H., & Mondal, B. (2012). Geochemical signature of provenance, tectonics and chemical weathering in the Quaternary flood plain sediments of the Hindon River, Gangetic plain, India. *Tectonophysics, 566*, 87–94.

[CR71] Müller, G. (1979). Schwermetalle in den sediments des rheins-veran-derngren seitt. 1971. *Umschan,**79*, 778–783.

[CR74] Pan, L., Ma, J., Wang, X., & Hou, H. (2016). Heavy metals in soils from a typical county in Shanxi Province, China: Levels, sources and spatial distribution. *Chemosphere,**148*, 248–254. 26807946 10.1016/j.chemosphere.2015.12.049

[CR75] Pehlivan, R. (2010). The effects of weathering in the Buyukmelen River basin on the geochemistry of suspended and bed sediments and the hyrogeochemical characteristics of river water, Duzce, Turkey. *Journal of Asian Earth Sciences,**39*, 62–75.

[CR76] Persaud, D., Jaagumagi, R., & Hayton, A. (1993). Guidelines for the protection and management of aquatic sediment quality in Ontario.

[CR77] Prabakaran, K., Nagarajan, R., Eswaramoorthi, S., Anandkumar, A., & Franco, F. M. (2018). Environmental significance and geochemical speciation of trace elements in Lower Baram River sediments. *Chemosphere,**219*, 933–953. ISSN 0045-6535. 30572242 10.1016/j.chemosphere.2018.11.158

[CR79] Prudêncio, M. I., Dias, M. I., Gouveia, M. A., Marques, R., Franco, D., & Trindade, M. J. (2009). Geochemical signatures of Roman amphorae produced in the Sado River estuary, Lusitania (Western Portugal). *Journal of Archaeological Science,**36*(3), 873–883.

[CR63] Rahman, M. S., Ahmed, Z., Seefat, S. M., et al. (2022a). Assessment of heavy metal contamination in sediment at the newly established tannery industrial estate in Bangladesh: A case study. *Environmental Chemistry and Ecotoxicology,**4*, 1–12.

[CR80] Rahman, M. S., Ahmed, Z., Seefat, S. M., Alam, R., Islam, A. R. M. T., Choudhury, T. R., Begum, B. A., & Idris, A. M. (2022b). Assessment of heavy metal contamination in sediment at the newly established tannery industrial estate in Bangladesh: A case study. *Environmental Chemistry and Ecotoxicology,**4*, 1–12. 10.1016/j.enceco.2021.10.001

[CR81] Rahman, M. A., Siddique, M. A. B., Khan, R., Reza, A. H. M. S., Khan, A. H. A. N., Akbor, M. A., Islam, M. S., Hasan, A. B., Hasan, M. I., & Elius, I. B. (2022c). Mechanism of arsenic enrichment and mobilization in groundwater from southeastern Bangladesh: Water quality and preliminary health risks assessment. *Chemosphere*. 10.1016/j.chemosphere.2022.133556 35007611

[CR82] Rahman, K., Barua, S., Ahammad, F., & Alam, M. A. (2020). Assessment of pollution and sources of heavy metals in the sediments of the Shitalakhya River, Bangladesh. *International Journal of Advanced Geosciences,**8*(1), 89–94. 10.14419/ijag.v8i1.30789

[CR83] Ramasamy, V., Paramasivam, K., Suresh, G., & Jose, M. T. (2014). Function of minerals in the natural radioactivity level of Vaigai River sediments, Tamilnadu, India—Spectroscopical approach. *Spectrochimica Acta Part a: Molecular and Biomolecular Spectroscopy,**117*, 340–350. 10.1016/j.saa.2013.08.022 24001975

[CR84] Rambabu, K., Avornyo, A., Gomathi, T., Thanigaivelan, A., Show, P. L., & Banat, F. (2023). Phytoremediation for carbon neutrality and circular economy: Potential, trends, and challenges. *Bioresource Technology,**367*, Article 128257. 36343781 10.1016/j.biortech.2022.128257

[CR85] Real, M. K. H., Khanam, N., Mia, M. Y., & Nasreen, M. (2017). Assessment of water quality and microbial load of Dhaleshwari River Tangail, Bangladesh. Advances in Microbiology,* 7*(06), 523.

[CR109] Resmi, M. R., & Achyuthan, H. (2018). Lower Palar river sediments, southern peninsular, India: Geochemistry, source-area weathering, provenance and tectonic setting. *Journal of the Geological Society of India, 92*(1), 83–91.

[CR86] Richards, L. A., Kumar, A., Shankar, P., Gaurav, A., Ghosh, A., & Polya, D. A. (2020). Distribution and geochemical controls of arsenic and uranium in groundwaterderived drinking water in Bihar, India. *International Journal of Environmental Research and Public Health,**17*(7), Article 2500. 10.3390/ijerph17072500 32268538 PMC7177302

[CR87] Rudnick, R. L., & Gao, S. (2014). Composition of the Continental Crust. Treatise on Geochemistry, second ed., pp. 1–64 (Chapter 4).

[CR89] Saha, T. K., & Pal, S. (2019). Exploring physical wetland vulnerability of Atreyee river basin in India and Bangladesh using logistic regression and fuzzy logic approaches. *Ecological Indicators,**98*, 251–265. 10.1016/j.ecolind.2018.11.009

[CR91] Sheikh, M. A., Noah, N. M., Tsuha, K., & Oomori, T. (2007). Occurrence of tributyltin compounds and characteristics of heavy metals. *International Journal of Environmental Science and Technology,**4*, 49–60.

[CR112] Siddiqui, E., & Pandey, J. (2020). Assessment of heavy metal pollution in water and surface sediment and evaluation of ecological risks associated with sediment contamination in the Ganga River: A basin-scale study. *Environmental Science and Pollution Research, 26*(11), 10926–10940. 10.1007/s11356-019-04495-6. 30783925

[CR92] Sorokina, O. A., & Zarubina, N. V. (2011). Chemical composition of the bottom sediments in the middle reaches of the Amur River. *Russian Journal of Pacific Geology,**5*(5), 469–476. 10.1134/S1819714011050095

[CR93] Tamim, U., Khan, R., Jolly, Y. N., Fatema, K., Das, S., Naher, K., Islam, M. A., Islam, S. M. A., & Hossain, S. M. (2016). Elemental distribution of metals in urban river sediments near an industrial effluent source. *Chemosphere,**155*, 509–518. 10.1016/j.chemosphere.2016.04.099 27151427

[CR94] Tuhin, A. K. (2025). CETP crisis chokes leather industry's growth. The Financial Express. https://thefinancialexpress.com.bd/views/columns/cetp-crisis-chokes-leather-industrys-growth

[CR97] Upadhyay, P., Ladumor, R., Gurjar, T., Kottayi, M., Doshi, A., & Pandya, P. (2025). Environmental risk assessment of heavy metals and microplastics in marine biota along Gujarat coastline, India. *Marine Pollution Bulletin,**211*, Article 117357. 39642433 10.1016/j.marpolbul.2024.117357

[CR98] Upadhyay, P., Patel, V., Gurjar, T., Ladumor, R., Kottayi, M., Doshi, A., Salunke, A., & Pandya, P. (2026). Exploring synergistic contamination of heavy metals and microplastics in marine edible fishes and associated risk status in humans. *Marine Pollution Bulletin,**222*, Article 118700. 40961569 10.1016/j.marpolbul.2025.118700

[CR110] Wu, W., Zheng, H., Xu, S., Yang, J., & Liu, W. (2013). Trace element geochemistry of riverbed and suspended sediments in the upper Yangtze River. *Journal of Geochemical Exploration, 124*, 67-78.

[CR121] Wu, H., Li, J., & Lin, A. (2026). Learning from residuals: A knowledge-data-driven hybrid model for explaining the social-ecological influences on urban agglomeration resilience. *Sustainable Cities and Society, 147*, 107539. 10.1016/j.scs.2026.107539

[CR99] Xiao, J., Wang, L., Deng, L., & Jin, Z. (2019a). Characteristics, sources, water quality and health risk assessment of trace elements in river water and well water in the Chinese Loess Plateau. *Science of the Total Environment,**650*, 2004–2012. 30290343 10.1016/j.scitotenv.2018.09.322

[CR118] Xiao, K., Ma, J., Hu, X., Dong, Y., & Tian, Y. (2019b). Influence of curing condition on properties of solidified clay in landfill leachate environment. *Environmental Progress & Sustainable Energy, 38*(5), 13149. 10.1002/ep.13149

[CR100] Xu, C., Huang, Y., Yang, B., Jia, R., Ning, Z., Yang, B., Xie, L., Yang, L., Lu, D., Kang, Z., & Guo, L. (2026). Molecular-weight-dependent mixing behavior of organic phosphorus and carbon in a subtropic river-estuary-bay continuum. *Water Research,**296*, Article 125650. 10.1016/j.watres.2026.125650 41775044

[CR101] Yu, W., Liu, R., & Wang, J. (2015). Source apportionment of PAHs in surface sediments using positive matrix factorization combined with GIS for the estuarine area of the Yangtze River, China. *Chemosphere,**134*, 263–271. 10.1016/j. 25966456 10.1016/j.chemosphere.2015.04.049

[CR116] Zhan, Y., Guo, Z., Podgorski, J., Zeng, Z., Xu, P., Peng, L., et al. (2025). Changes in meat consumption can improve groundwater quality. *Nature Food, 6*(7), 703-714. 10.1038/s43016-025-01188-x 40514542

[CR114] Zhang, Q., Zhang, K., Chao, L., Chen, X., & Wu, N. (2024). A unified runoff generation scheme for applicability across different hydrometeorological zones. *Environmental Modelling & Software, 180*, 106138. 10.1016/j.envsoft.2024.106138

[CR102] Zhang, J., Geng, Y., Li, Z., Li, P., Wang, T., Xu, G., et al. (2025a). Modeling sediment flux in river confluences: A comprehensive momentum-based study. *Water Resources Research,**61*, Article e2024WR039154. 10.1029/2024WR039154

[CR113] Zhang, Q., Zhang, K., Bárdossy, A., Li, Y., & Wu, N. (2025b). Improving representation of hydrological process heterogeneity in grid-Xin’anjiang model through a stepwise approach. *Journal of Hydrology, 655*, 132897. 10.1016/j.jhydrol.2025.132897

[CR103] Zhao, M. Y., & Zhen, Y. F. (2015). The intensity of chemical weathering: Geochemical constraints from marine detrital sediments of Triassic age in South China. *Chemical Geology,**391*, 111–122. 10.1016/j.chemgeo.2014.11.004

[CR104] Zheng, N., Wang, Q., Liang, Z., & Zheng, D. (2008). Characterization of heavy metal concentrations in the sediments of three freshwater rivers in Huludao City, Northeast China. *Environmental Pollution,**154*, 135–142. 18280624 10.1016/j.envpol.2008.01.001

[CR120] Zhi, L., Shao, D., Xie, X., Li, X., Cui, B., et al. (2025). Natural process-based coastal wetland restoration by relocating river flow paths in a river-dominated delta. *Communications Earth & Environment, 6*(1), 608. 10.1038/s43247-025-02612-7

[CR119] Zhi, L., Li, X., Li, X., Ma, T., Guo, W., Cui, B., et al. (2026). An integrated strategy maximises cobenefits of conservation and restoration for ecosystem services in coastal wetlands. *Communications Earth & Environment, 7*(1), 363. 10.1038/s43247-026-03376-4

[CR122] Zhuo, T., You, X., He, L., Chai, B., Anthony, M., Benocci, T., et al. (2026). Stratification enhances yet self-limits carbon sequestration through microbial necromass accumulation at reservoir sediment–water interfaces. *Journal of Cleaner Production, 572*, 148911. 10.1016/j.jclepro.2026.148911

[CR105] Zvinowanda, C. M., Okonkwo, J. O., Shabalala, P. N., & Agyei, N. M. (2009). A novel adsorbent for heavy metal remediation in aqueous environments. *International Journal of Environmental Science and Technology,**6*, 425–434.

