# Supplementary material for: Chromium as a severe anthropogenic geochemical anomaly in river sediments near a tannery outfall: spatial attenuation, source apportionment and ecological risk screening
Source: Environ Geochem Health. 2026 Sep 3;48(14):570. doi: 10.1007/s10653-026-03457-5 (PMC13541875; doi:10.1007/s10653-026-03457-5)
Supplement: Supplementary file 1 [file 10653_2026_3457_MOESM1_ESM.docx]

**Chromium as a severe anthropogenic geochemical anomaly in river sediments near a tannery outfall: spatial attenuation, source apportionment and ecological risk screening**

Rahat Khan^1,^*, Shuvashish Das Shuvo^1, 2^, Md. Samium Basir^1, 2^, Md. Abu Bakar Siddique^3^, **Javed Mallick^4^,** Hoang Thi Hang^4^, Md. Harunor Rashid Khan^5^, Saad Aldawood^6^, Dhiman Kumer Roy^7^, Narottam Saha^8,^*

^1^Institute of Nuclear Science & Technology, Bangladesh Atomic Energy Commission (BAEC), Savar, Dhaka 1349, Bangladesh

^2^Department of Environmental Science, Bangladesh University of Professionals (BUP), Mirpur-12, Cantonment, Dhaka 1216, Bangladesh

*^3^*Institute of National Analytical Research and Service (INARS), Bangladesh Council of Scientific and Industrial Research (BCSIR), Dhanmondi, Dhaka 1205, Bangladesh

^4^Department of Civil Engineering, College of Engineering, King Khalid University, Abha, Kingdom of Saudi Arabia

^5^Physics Discipline, Khulna University, Khulna 9208, Bangladesh

^6^Department of Physics and Astronomy, College of Science, P.O. BOX 2455, King Saud University, Riyadh 11451, Saudi Arabia

^7^Department of Geology and Mining, University of Barishal, Barishal 8254, Bangladesh

^8^Center for Mined Land Rehabilitation, Sustainable Minerals Institute, The University of Queensland, Saint Lucia, QLD 4072, Australia

*Corresponding author.

Email address: [rahatkhan.baec@gmail.com](mailto:rahatkhan.baec@gmail.com) (Rahat Khan); [n.saha@uq.edu.au](mailto:n.saha@uq.edu.au) (Narottam Saha)

**Table S1:** Sample Information with ancillary data.

| Location | Sample ID | Depth from the surface [cm] | Note |
| --- | --- | --- | --- |
| S-1 | 1.1 | 0-5 | Discharge Point of Tannery Industry |
|  | 1.2 | 5-10 |  |
|  | 1.3 | 10-15 |  |
|  | 1.4 | 15-20 |  |
|  | 1.5 | 20-25 |  |
| S-2 | 2.1 | 0-5 | Sampling point in 50m distance with 45º North-West direction from discharge point |
|  | 2.2 | 5-10 |  |
|  | 2.3 | 10-15 |  |
| S-3 | 3.1 | 0-5 | Sampling point in 50m distance with 90º West direction from discharge point |
|  | 3.2 | 5-10 |  |
|  | 3.3 | 10-15 |  |
| S-4 | 4.1 | 0-5 | Sampling point in 50m distance with 45º South-West direction from discharge point |
|  | 4.2 | 5-10 |  |
|  | 4.3 | 10-15 |  |
| S-5 | 5.1 | 0-5 | Sampling point in 100m distance with 45º North-West direction from discharge point |
|  | 5.2 | 5-10 |  |
|  | 5.3 | 10-15 |  |
| S-6 | 6.1 | 0-5 | Sampling point in 100m distance with 90º West direction from discharge point |
|  | 6.2 | 5-10 |  |
|  | 6.3 | 10-15 |  |
| S-7 | 7.1 | 0-5 | Sampling point in 100m distance with 45º South-West direction from discharge point |
|  | 7.2 | 5-10 |  |
|  | 7.3 | 10-15 |  |
| S-8 | 8.1 | 0-5 | Sampling point in 150m distance with 45º North-West direction from discharge point |
|  | 8.2 | 5-10 |  |
|  | 8.3 | 10-15 |  |
| S-9 | 9.1 | 0-5 | Sampling point in 150m distance with 90º West direction from discharge point |
|  | 9.2 | 5-10 |  |
|  | 9.3 | 10-15 |  |
| S-10 | 10.1 | 0-5 | Sampling point in 150m distance with 45º South-West direction from discharge point |
|  | 10.2 | 5-10 |  |
|  | 10.3 | 10-15 |  |
| S-11 | 11 | 0-15 | Sampling point in 370m distance with 45º North-West direction from discharge point |
| S-12 | 12 | 0-15 | Sampling point in 400m distance with 90º West direction from discharge point |
| S-13 | 13 | 0-15 | Sampling point in 420m distance with 45º South-West direction from discharge point |
| S-14 | 14.1 | 0-5 | Sampling point with 1500m distance in upstream direction from discharge point |
|  | 14.2 | 5-10 |  |
|  | 14.3 | 10-15 |  |
|  | 14.4 | 15-20 |  |
|  | 14.5 | 20-25 |  |
| S-15 | 15.1 | 0-5 | Sampling point with 50 m distance in upstream direction from discharge point |
|  | 15.2 | 5-10 |  |
|  | 15.3 | 10-15 |  |
|  | 15.4 | 15-20 |  |
|  | 15.5 | 20-25 |  |
| S-16 | 16.1 | 0-5 | Sampling point with 1.5 km distance in downstream direction from discharge point |
|  | 16.2 | 5-10 |  |
|  | 16.3 | 10-15 |  |
|  | 16.4 | 15-20 |  |
|  | 16.5 | 20-25 |  |
| S-17 | 17.1 | 0-5 | Sampling point with 2 km distance in downstream direction from discharge point |
|  | 17.2 | 5-10 |  |
|  | 17.3 | 10-15 |  |
|  | 17.4 | 15-20 |  |
|  | 17.5 | 20-25 |  |
| S-18 | 18.1 | 0-5 | Sampling point with 2.5 km distance in downstream direction from discharge point |
|  | 18.2 | 5-10 |  |
|  | 18.3 | 10-15 |  |
|  | 18.4 | 15-20 |  |
|  | 18.5 | 20-25 |  |

**Table S2:** Descriptive statistics of elemental abundances in repeated analyses (n = 3) of IAEA-RM-Soil-7 (soil) and IAEA-RM-SL-1 (lake sediment) of this study along with the certificate values, detection limits (DL: 3σ), measured radionuclides with their half-lives.

|  |  | IAEA-RM-Soil-7 (soil) | | | | | | |  | IAEA-RM-SL-1 (lake sediment) | | | | | | |  | Measured | Half- | Counting | Gamma energy (keV) | DL (2σ) |
| --- | --- | --- | --- | --- | --- | --- | --- | --- | --- | --- | --- | --- | --- | --- | --- | --- | --- | --- | --- | --- | --- | --- |
|  |  | This work | | |  | Certificate | | |  | This work | | |  | Certificate | | |  | Radionuclide | life | mood |  |  |
|  |  | Mean | SD | RSD |  | Value | Min. | Max. |  | Mean | SD | RSD |  | Value | Min. | Max. |  |  |  |  |  |  |
| Elements | Unit | (n=3) | (1σ) | (%) |  |  |  |  |  | (n=3) | (1σ) | (%) |  |  |  |  |  |  |  |  |  |  |
| Na | [%] | 0.231 | 0.012 | 5.2 |  | *0.24* | *0.23* | *0.25* |  | 0.165 | 0.012 | 7.3 |  | 0.170 | 0.160 | 0.180 |  | ^24^Na | 15.02h | S2 | 1368.8, 2754 | 0.001 |
| Al | [%] | 4.86 | 0.18 | 3.7 |  | *4.7* | *4.4* | *5.1* |  | 9.45 | 0.72 | 7.6 |  | *8.9* |  |  |  | ^28^Al | 2.25m | S1 | 1778.80 | 0.004 |
| K | [%] | 1.22 | 0.06 | 4.9 |  | *1.21* | *1.13* | *1.27* |  | 1.35 | 0.06 | 4.4 |  | 1.45 | 1.24 | 1.66 |  | ^42^K | 12.36h | S2 | 1524.60 | 0.034 |
| Ti | [%] | 0.338 | 0.028 | 8.3 |  | *0.30* | *0.26* | *0.37* |  | 0.489 | 0.048 | 9.8 |  | 0.517 | 0.474 | 0.560 |  | ^51^Ti | 5.76m | S1 | 320.10 | 0.026 |
| Cr | [µg/g] | 64 | 3 | 4.7 |  | 60 | 49 | 74 |  | 106 | 8 | 7.5 |  | *104* | *95* | *113* |  | ^51^Cr | 27.7 d | L3 | 320.10 | 1.30 |
| Mn | [µg/g] | 640 | 20 | 3.1 |  | 631 | 604 | 650 |  | 3520 | 50 | 1.4 |  | 3460 | 3300 | 3620 |  | ^56^Mn | 2.58m | S2 | 846.6, 1810.7, 2113.2 | 0.40 |
| Co | [µg/g] | 9.34 | 0.51 | 5.5 |  | 8.9 | 8.4 | 10.1 |  | 18.7 | 1.1 | 5.9 |  | 19.8 | 18.3 | 21.3 |  | ^60^Co | 5.27y | L3 | 1173.2, 1332.5 | 0.130 |
| Zn | [µg/g] | 105 | 9 | 8.6 |  | 104 | 101 | 113 |  | 227 | 18 | 7.9 |  | 223 | 213 | 233 |  | ^65^Zn | 244.1 d | L3 | 1115.50 | 3.160 |
| As | [µg/g] | 13.7 | 1.2 | 8.8 |  | 13.4 | 12.5 | 14.2 |  | 28.4 | 0.8 | 2.8 |  | 27.6 | 24.7 | 30.5 |  | ^76^As | 26.30h | L1 | 559.10 | 0.190 |
| Rb | [µg/g] | 52 | 4 | 7.7 |  | 51 | 47 | 56 |  | 107 | 9 | 8.4 |  | 113 | 102 | 124 |  | ^86^Rb | 18.66d | L2 | 1076.7 | 3.10 |
| Sb | [µg/g] | 1.65 | 0.15 | 9.1 |  | 1.7 | 1.4 | 1.8 |  | 1.21 | 0.10 | 8.3 |  | *1.31* | *1.19* | *1.43* |  | ^124^Sb | 2.75d | L1 | 564.20 | 0.05 |
| Cs | [µg/g] | 5.18 | 0.31 | 6.0 |  | 5.4 | 4.9 | 6.4 |  | 6.87 | 0.50 | 7.3 |  | *7.0* | *6.1* | *7.9* |  | ^134^Cs | 2.062y | L3 | 604.7, 795.8 | 0.50 |
| Ba | [µg/g] | 144 | 9 | 6.3 |  | *159* | *131* | *196* |  | 622 | 40 | 6.4 |  | 639 | 586 | 692 |  | ^131^Ba | 11.8d | L2 | 123.8, 216, 373.2, 496.3 | 32.0 |
| Th | [µg/g] | 7.87 | 0.65 | 8.3 |  | 8.2 | 6.5 | 8.7 |  | 13.2 | 0.9 | 6.8 |  | 14 | 13 | 15 |  | ^233^Pa | 27 d | L3 | 311.9 | 0.066 |
| U | [µg/g] | 2.97 | 0.22 | 7.4 |  | 2.6 | 2.2 | 3.3 |  | 3.95 | 0.22 | 5.6 |  | *4.02* | *3.69* | *4.35* |  | ^239^Np | 2.35d | L1 | 106.1, 228.2, 277 | 0.10 |

IAEA-RM-Soil-7: Certificate values are taken from IAEA reference sheet issued on January, 2000. IAEA-RM-SL-1: Certificate values for all the elements are taken from IAEA reference sheet issued on September, 1999. All the values are recommended values; except for Italics are information values only. Decay data (here half-life) of the measured radionuclides are taken from Randa et al. (2007), except for ^122^Sb (Khan et al., 2019) and ^58^Co (Hall et al., 1990).

**Table S3**: Mathematical equations with the importance of geo-environmental and ecological indices.

|  | Indices | Mathematical equations | Classifications | Contamination degree | References |
| --- | --- | --- | --- | --- | --- |
| Geo-environmental indices | Geo-accumulation index (*I_geo_*) | *I_geo_= log_2_* $(\frac{C_{n}}{1.5\times B_{n}}$*)* Where, *C_n_* is the measured elemental concentration in sediment and *B_n_* is the background heavy metal concentrations | I_geo_<0  0≤I_geo_<1  1≤I_geo_<2  2≤I_geo_<3  3≤I_geo_<4  4≤I_geo_<5  I_geo_≥5 | Practically uncontaminated  Uncontaminated to moderately contaminated  Moderately contaminated  Moderately to heavily contaminated  Heavily contaminated  Heavily to extremely contaminated  Extremely contaminated | Müller (1979); Kumar et al. (2022); Kumar et al. (2021) |
|  | Contamination factor (*CF*) | *CF_metals_=* $\frac{C_{n}}{B_{n}}$ Where, *C_n_* and *B_n_* are the measured and background elemental concentrations in sediment respectively | CF<1  1≤CF<3  3≤CF<6  CF≥6 | Low contamination  Moderate contamination  Considerable contamination  Very high contamination | Hakanson (1980); Kumar et al. (2022); Kumar et al. (2021) |
|  | Degree of contamination (*C_d_*) | *C_d_ =*$\sum_{i=1}^{n} {CF}_{i}$ where, CF is the contamination factor | C_d_<8  8≤C_d_<16  16≤C_d_<32  C_d_≥32 | Low contamination  Moderate contamination  Considerable contamination  Very high contamination | Kumar et  al. (2022); Hakanson (1980) |
|  | Modified degree of contamination (*mC_d_*) | mC_d_$=\frac{1}{n}\sum_{i=1}^{n} {CF}_{i}$  where, CF is the contamination factor | mC_d_<1.5  1.5≤mC_d_<2  2≤mC_d_<4  4≤mC_d_<8  8≤mC_d_<16  16≤mC_d_<32  mC_d_>32 | Nil to very low contamination  Low contamination  Moderate contamination  High contamination  Very high contamination  Extremely high contamination  Ultra high contamination | Abrahim and Parker (2008); Kumar et al. (2021); Kumar et al. (2022) |
|  | Enrichment factor (*EF*) | EF=$\frac{\left( \frac{C_{n}}{Al} \right)sample}{\left( \frac{B_{n}}{Al} \right)background}$  Where, *C_n_* is the measured elemental concentration in sediment and *B_n_* is the background heavy metal concentrations | EF = 1  1 ≤ EF < 1.5  1.5 ≤ EF < 3  3 ≤ EF < 5  5 ≤ EF < 10  EF > 10 | Baseline data  Possible anthropogenic origin  Minor enrichment  Moderate enrichment  Severe modification  Very severe modification | Tamim et al. (2016); Kumar et al. (2022); Islam et al. (2017) |
|  | Pollution load index (*PLI*) | PLI=$\sqrt[n]{(CF_{1}\times CF_{2} \times\ldots CF_{n})}$ where, CF is the contamination factor | PLI = 0  PLI < 1  PLI > 1 | Perfection  Baseline level  Contaminated | Kumar et al. (2022); Kumar et al. (2021) |
| Sediment quality guideline-based indices | Mean ERM Quotient (*M-ERM-Q*) | M-ERM-Q = $\frac{\sum_{i=1}^{n} \frac{C_{i}}{{ERM}_{i}}}{n}$  where, *C_i_* is the concentration of element i, *ERM_i_* is the ERM values for the element i and *n* is the number of elements | M-ERM-Q < 0.1  0.11 ≤ M-ERM-Q < 0.5  0.51 ≤ M-ERM-Q < 1.5  M-ERM-Q > 1.5 | 9% probability of toxicity  21% probability of toxicity  49% probability of toxicity  76% probability of toxicity | Long et al. (2000); Islam et al. (2017) |
|  | Toxic Unit analysis (*TU*) | *TU=* $\frac{C_{M}}{PEL}$ where, *C_M_* is the concentration of heavy metal and *PEL* is the probable effect level.  *∑ TUs = TU_metal1 +_ TU_metal2 +_ TU_metal3_ ………+ TU_metaln_* where, *ΣTUs* is the sum of toxic units for metals in sediments | ΣTUs < 4  4 ≤ ΣTUs ≤ 6  ΣTUs > 6 | Low toxicity level  Moderate toxicity level  Heavy toxicity level | Islam et al. (2020); Kumar et al. (2021) |
|  | Environmental Toxicity Quotient (ETQ) | ETQ = $\frac{\sum_{i=1}^{n} TS_{i}\times\frac{C_{i}}{{TS}_{As}}}{n}$  where, *C_i_* is the concentration of element i,and n is the number of analyzed elements. TS*_i_* is the total score of each element and TS_As_ is the total score of arsenic published by Agency for Toxic Substances and Disease Registry (ASTDR). The ASTDR provided scores for each element are As = 1674, Pb = 1531, Cd = 1320, Co = 1013, Ni = 996, Zn = 915, Cr = 895, Cu = 807 | ETQ < 10  10 ≤ ETQ ≤ 50  50 ≤ ETQ ≤ 100  100 ≤ ETQ ≤ 300  ETQ >300 | Low toxicity level  Moderate toxicity level  High toxicity level  Very High toxicity level  Extremely High toxicity level | Maftei et al. (2019); Ali et al. (2018); ASTDR (2017) |

**Table S4**: Normality test of elemental variables in Dhaleshwari River, Bangladesh.

|  | Kolmogorov-Smirnov^a^ | | | Shapiro-Wilk |  |  |
| --- | --- | --- | --- | --- | --- | --- |
|  | Statistic | df | Sig. | Statistic | df | Sig. |
| Na | 0.251 | 60 | 0.000 | 0.751 | 60 | 0.000 |
| Al | 0.143 | 60 | 0.004 | 0.898 | 60 | 0.000 |
| K | 0.202 | 60 | 0.000 | 0.788 | 60 | 0.000 |
| Ti | 0.160 | 60 | 0.001 | 0.933 | 60 | 0.003 |
| Cr | 0.396 | 60 | 0.000 | 0.366 | 60 | 0.000 |
| Mn | 0.080 | 60 | .200^*^ | 0.980 | 60 | 0.425 |
| Co | 0.139 | 60 | 0.006 | 0.944 | 60 | 0.009 |
| Zn | 0.265 | 60 | 0.000 | 0.686 | 60 | 0.000 |
| As | 0.083 | 60 | .200^*^ | 0.966 | 60 | 0.095 |
| Rb | 0.149 | 60 | 0.002 | 0.919 | 60 | 0.001 |
| Sb | 0.137 | 60 | 0.007 | 0.881 | 60 | 0.000 |
| Cs | 0.101 | 60 | .200^*^ | 0.965 | 60 | 0.085 |
| Ba | 0.131 | 60 | 0.012 | 0.971 | 60 | 0.159 |
| Th | 0.157 | 60 | 0.001 | 0.865 | 60 | 0.000 |
| U | 0.088 | 60 | .200^*^ | 0.949 | 60 | 0.014 |
| *. This is a lower bound of the true significance. |  |  |  |  |  |  |
| a. Lilliefors Significance Correction |  |  |  |  |  |  |

**Table S5**: Normality test after elemental variables transform in Dhaleshwari River, Bangladesh.

|  | Kolmogorov-Smirnov^a^ | | | Shapiro-Wilk |  |  |
| --- | --- | --- | --- | --- | --- | --- |
|  | Statistic | df | Sig. | Statistic | df | Sig. |
| Na | 0.211 | 60 | 0.000 | 0.835 | 60 | 0.000 |
| Al | 0.197 | 60 | 0.000 | 0.690 | 60 | 0.000 |
| K | 0.296 | 60 | 0.000 | 0.556 | 60 | 0.000 |
| Ti | 0.202 | 60 | 0.000 | 0.904 | 60 | 0.000 |
| Cr | 0.176 | 60 | 0.000 | 0.892 | 60 | 0.000 |
| Mn | 0.096 | 60 | .200^*^ | 0.952 | 60 | 0.019 |
| Co | 0.175 | 60 | 0.000 | 0.898 | 60 | 0.000 |
| Zn | 0.160 | 60 | 0.001 | 0.893 | 60 | 0.000 |
| As | 0.107 | 60 | 0.088 | 0.933 | 60 | 0.003 |
| Rb | 0.197 | 60 | 0.000 | 0.761 | 60 | 0.000 |
| Sb | 0.131 | 60 | 0.012 | 0.963 | 60 | 0.066 |
| Cs | 0.182 | 60 | 0.000 | 0.828 | 60 | 0.000 |
| Ba | 0.169 | 60 | 0.000 | 0.937 | 60 | 0.004 |
| Th | 0.205 | 60 | 0.000 | 0.854 | 60 | 0.000 |
| U | 0.065 | 60 | .200^*^ | 0.980 | 60 | 0.443 |
| *. This is a lower bound of the true significance. |  |  |  |  |  |  |
| a. Lilliefors Significance Correction |  |  |  |  |  |  |

**Table S6:** Non parametric test of elemental variables in Dhaleshwari River, Bangladesh.

|  | Na | Al | K | Ti | Cr | Mn | Co | Zn | As | Rb | Sb | Cs | Ba | Th | U |
| --- | --- | --- | --- | --- | --- | --- | --- | --- | --- | --- | --- | --- | --- | --- | --- |
| Test Value^a^ | 13000.00 | 73500.00 | 22000.00 | 4000.00 | 373.00 | 601.00 | 16.30 | 105.50 | 4.20 | 135.50 | 1.20 | 8.30 | 456.50 | 18.15 | 3.90 |
| Cases < Test Value | 19 | 30 | 24 | 19 | 30 | 30 | 30 | 30 | 30 | 30 | 29 | 30 | 30 | 30 | 29 |
| Cases >= Test Value | 41 | 30 | 36 | 41 | 30 | 30 | 30 | 30 | 30 | 30 | 31 | 30 | 30 | 30 | 31 |
| Total Cases | 60 | 60 | 60 | 60 | 60 | 60 | 60 | 60 | 60 | 60 | 60 | 60 | 60 | 60 | 60 |
| Number of Runs | 18 | 24 | 18 | 14 | 12 | 21 | 18 | 23 | 23 | 23 | 22 | 18 | 27 | 23 | 22 |
| Z | -2.705 | -1.823 | -3.203 | -3.912 | -4.948 | -2.604 | -3.385 | -2.083 | -2.083 | -2.083 | -2.338 | -3.385 | -1.042 | -2.083 | -2.338 |
| Asymp. Sig. (2-tailed) | 0.007 | 0.068 | 0.001 | 0.000 | 0.000 | 0.009 | 0.001 | 0.037 | 0.037 | 0.037 | 0.019 | 0.001 | 0.298 | 0.037 | 0.019 |
| a. Median |  |  |  |  |  |  |  |  |  |  |  |  |  |  |  |

**Table S7**. Spatial statistics or Cr versus distance from the tannery discharge point (S-1).

| **Statistic** | **Result** |
| --- | --- |
| Spearman correlation, Cr vs. flow-axis distance from S-1 (n = 30; sites S-1, S-14 to S-18) | ρ = -0.383, p = 0.036 |
| OLS regression, log10(Cr) ~ flow-axis distance (same subset) | slope = -0.00022 log10 units/m, R² = 0.125, p = 0.055 |
| Kruskal-Wallis, Cr by Zone — site-level data (n = 18 independent sites) | χ^2^ = 10.89, df = 3, p = 0.012 |
| Dunn's post-hoc (Bonferroni-adjusted), Discharge zone vs. Downstream (site-level) | p = 0.024 |
| Dunn's post-hoc (Bonferroni-adjusted), Discharge zone vs. Upstream (site-level) | p = 1.000 |
| Dunn's post-hoc (Bonferroni-adjusted), Discharge zone vs. Opposite riverbank (site-level) | p = 0.159 |

**Table S8**. Depth-wise (vertical) statistics for Cr.

| Statistic | Result |
| --- | --- |
| Friedman test, Cr across 5 depth layers — full-profile sites (S-1, S-14 to S-18; n = 6 sites) | χ² = 2.53, df = 4, p = 0.639 |
| Friedman test, Cr across 3 depth layers — near-field sites (S-2 to S-10; n = 9 sites) | χ² = 6.89, df = 2, p = 0.032 |
| Wilcoxon signed-rank, surface vs. deepest layer (paired by site, n = 15 core sites) | V = 99, p = 0.027; median surface = 1056 µg/g, median deepest = 237 µg/g |

Note: Friedman's test blocks by site, so only within-core rank order across depth is compared; concentration magnitude is never compared between unrelated sites.

**Table S9**: Varimax rotated principal component analysis of elemental variables in Dhaleshwari River, Bangladesh.

| **Elements** | **Principal Component** | | |
| --- | --- | --- | --- |
|  | **PC1** | **PC2** | **PC3** |
| Na | -0.160 | **-0.862** | 0.044 |
| Al | **0.736** | 0.601 | 0.142 |
| K | **0.623** | 0.702 | 0.190 |
| Ti | **0.764** | 0.260 | 0.106 |
| Cr | -0.293 | **-0.911** | -0.172 |
| Mn | **0.747** | 0.149 | 0.280 |
| Co | **0.940** | 0.163 | 0.155 |
| Zn | -0.031 | **-0.719** | -0.314 |
| As | **0.827** | -0.181 | 0.153 |
| Rb | **0.809** | 0.491 | 0.186 |
| Sb | -0.367 | 0.134 | -0.606 |
| Cs | **0.864** | 0.301 | 0.135 |
| Ba | **0.710** | 0.350 | 0.318 |
| Th | 0.028 | 0.343 | **0.816** |
| U | 0.277 | 0.201 | **0.748** |
| **Eigenvalues** | 8.304 | 2.038 | 1.319 |
| **% of Variance** | 55.363 | 13.590 | 8.796 |
| **Cumulative %** | 55.363 | 68.952 | 77.749 |

Extraction Method: Principal Component Analysis.

Rotation Method: Varimax with Kaiser Normalization

**Table S10:** Source apportioning contributions of metal(oid)s in Dhaleshwari River sediment by the PMF model.

| **Element** |  | **Factor 1** |  | **Factor 2** |  | **Factor 3** |
| --- | --- | --- | --- | --- | --- | --- |
| **Na** |  | 29.97 |  | **32.32** |  | **37.71** |
| **Al** |  | **75.95** |  | 18.07 |  | 5.98 |
| **K** |  | **66.72** |  | 30.71 |  | 2.57 |
| **Ti** |  | **81.70** |  | 2.72 |  | 15.59 |
| **Cr** |  | 33.53 |  | **46.06** |  | 20.41 |
| **Mn** |  | 68.97 |  | 17.14 |  | 13.89 |
| **Co** |  | **85.22** |  | 0.00 |  | 14.78 |
| **Zn** |  | 43.79 |  | **19.90** |  | 36.30 |
| **As** |  | **75.89** |  | 0.00 |  | 24.11 |
| **Rb** |  | **77.99** |  | 14.66 |  | 7.35 |
| **Sb** |  | 44.59 |  | 0.24 |  | **55.18** |
| **Cs** |  | **89.27** |  | 0.00 |  | 10.73 |
| **Ba** |  | 62.39 |  | **21.59** |  | 16.02 |
| **Th** |  | **71.05** |  | 19.53 |  | 9.42 |
| **U** |  | **75.74** |  | 9.28 |  | 14.97 |

**Table S11:** Correlations among the analyzed parameters for Dhaleshwari River sediment, Bangladesh.

| **Parameters** | **Na** | **Al** | **K** | **Ti** | **Cr** | **Mn** | **Co** | **Zn** | **As** | **Rb** | **Sb** | **Cs** | **Ba** | **Th** | **U** |
| --- | --- | --- | --- | --- | --- | --- | --- | --- | --- | --- | --- | --- | --- | --- | --- |
| **Na** | 1 |  |  |  |  |  |  |  |  |  |  |  |  |  |  |
| **Al** | -.624^**^ | 1 |  |  |  |  |  |  |  |  |  |  |  |  |  |
| **K** | -.640^**^ | .937^**^ | 1 |  |  |  |  |  |  |  |  |  |  |  |  |
| **Ti** | -.335^**^ | .708^**^ | .611^**^ | 1 |  |  |  |  |  |  |  |  |  |  |  |
| **Cr** | .834^**^ | -.777^**^ | -.864^**^ | -.491^**^ | 1 |  |  |  |  |  |  |  |  |  |  |
| **Mn** | -0.147 | .631^**^ | .618^**^ | .681^**^ | -.448^**^ | 1 |  |  |  |  |  |  |  |  |  |
| **Co** | -.280^*^ | .792^**^ | .697^**^ | .770^**^ | -.428^**^ | .764^**^ | 1 |  |  |  |  |  |  |  |  |
| **Zn** | .476^**^ | -.439^**^ | -.558^**^ | -.353^**^ | .714^**^ | -.362^**^ | -0.215 | 1 |  |  |  |  |  |  |  |
| **As** | -0.043 | .490^**^ | .388^**^ | .600^**^ | -0.137 | .689^**^ | .727^**^ | -0.041 | 1 |  |  |  |  |  |  |
| **Rb** | -.510^**^ | .921^**^ | .895^**^ | .720^**^ | -.703^**^ | .659^**^ | .890^**^ | -.387^**^ | .540^**^ | 1 |  |  |  |  |  |
| **Sb** | 0.023 | -.291^*^ | -.293^*^ | -.343^**^ | 0.155 | -.327^*^ | -.382^**^ | 0.143 | -.401^**^ | -.327^*^ | 1 |  |  |  |  |
| **Cs** | -.440^**^ | .850^**^ | .764^**^ | .669^**^ | -.503^**^ | .604^**^ | .902^**^ | -0.192 | .631^**^ | .885^**^ | -.310^*^ | 1 |  |  |  |
| **Ba** | -.327^*^ | .769^**^ | .800^**^ | .489^**^ | -.551^**^ | .598^**^ | .779^**^ | -.275^*^ | .470^**^ | .881^**^ | -.320^*^ | .815^**^ | 1 |  |  |
| **Th** | -0.242 | .312^*^ | .387^**^ | 0.206 | -.450^**^ | .385^**^ | 0.221 | -.462^**^ | 0.139 | .339^**^ | -.259^*^ | 0.238 | .428^**^ | 1 |  |
| **U** | -.258^*^ | .489^**^ | .429^**^ | .318^*^ | -.347^**^ | .294^*^ | .421^**^ | -0.229 | 0.240 | .487^**^ | -.412^**^ | .507^**^ | .504^**^ | .589^**^ | 1 |
| **. Correlation is significant at the 0.01 level (2-tailed). | | | | | | | | | | | | | | | |
| *. Correlation is significant at the 0.05 level (2-tailed). | | | | | | | | | | | | | | | |

**Fig. S1. Distribution of Cr by spatial zone.**

**Fig. S2**: Spatial Distribution of 15 elements in Dhalehwari River Sediment

**Fig. S3**: Principal component analysis of the analyzed trace metal(oid)s by component plot in rotated space.

**Fig. S4**: Contribution of 3 apportioning factors for each element determined by the positive matrix factorization model.

**References**

Abrahim, G., Parker, R., 2008. Assessment of heavy metal enrichment factors and the degree of contamination in marine sediments from Tamaki Estuary, Auckland, New Zealand. Environ. Monit. Assess. 136 (1–3), 227–238.

Ali, M.M., Ali, M.L., Islam, M.S., Rahman, M.Z., 2018. Assessment of toxic metals in water and sediment of Pasur River in Bangladesh. Water Sci Technol 77:1418–1430. https://doi.org/10.2166/wst.2018.016

ASTDR, 2017. The Priority List of Hazardous Substances; ASTDR: Atlanta, GA, USA.

Hakanson, L. 1980. An ecological risk index for aquatic pollution control- A sedimentological approach. Water Res., 14(8): 975–1001

Hall, G.E.M., Bonham-Carter, G.F., MacLaurin, A.I., Ballantyne, S.B., 1990. Comparison of instrumental neutron activation analysis of geological materials with other multielement techniques. Talanta, 37(1), 135-155. https://doi: 10.1016/0039-9140(90)80054-j.

Islam, A.R.M.T., Hasanuzzaman, M., Islam, H.M.T., Mia, M.U., Khan, R., Habib, M.A., Rahman, M.M., Siddique, M.A.B., Moniruzzaman, M., Rashid, M.B., 2020. Quantifying Source Apportionment, Co‐occurrence, and Ecotoxicological Risk of Metals from Upstream, Lower Midstream, and Downstream River Segments, Bangladesh. Environmental Toxicology and Chemistry, 0:pp-1-14. http://dx.doi.org/10.1002/etc.4814

Islam, M.A., Al-mamun, A., Hossain, F., Quraishi, S.B., Naher, K., Khan, R., Das, S., Tamim, U., Hossain, S.M., Nahid, F., 2017. Contamination and ecological risk assessment of trace elements in sediments of the rivers of Sundarban mangrove forest, Bangladesh. Marine Pollution Bulletin 124: 356–366. http://dx.doi.org/10.1016/j.marpolbul.2017.07.059

Kumar, S., Islam, A.R.M.T., Hasanuzzaman, M., Salam, R., Islam, M.S., Khan, R., Rahman, M.S., Pal, S.C., Ali, M.M., Idris, A.M., Gustave, W., Elbeltagi, A., 2022. Potentially toxic elemental contamination in Wainivesi River, Fiji impacted by gold‑mining activities using chemometric tools and SOM analysis. Environmental Science and Pollution Research, https://doi.org/10.1007/s11356-022-18734-w

Kumar, S., Islam, A.R.M.T., Hasanuzzaman, M., Salam, R., Khan, R., Islam, M.S., 2021a. Preliminary assessment of heavy metals in surface water and sediment in Nakuvadra-Rakiraki River, Fiji using indexical and chemometric approaches. Journal of Environmental Management, 298:113517. https://doi.org/10.1016/j.jenvman.2021.113517

Kumar, S., Islam, A.R.M.T., Islam, H.M.T., Hasanuzzaman, M., Ongoma, V., Khan, R., Mallick, J., 2021b. Water resources pollution associated with risks of heavy metals from Vatukoula Goldmine region, Fiji. Journal of Environmental Management, 293: 112868. https://doi.org/10.1016/j.jenvman.2021.112868

Khan, R., Islam, M.S., Tareq, A.R.M., Naher, K., Islam, A.R.M,T., Habib, M.A., Siddique, M.A.B., Islam ,M.A., Das, S., Rashid, M.B., Ullah, A.K.M.A., Miah, M.M.H., Masrura, S.U., Bodrud-Doza, M., Sarker, M.R., Badruzzaman, A.B.M., 2020. Distribution, sources and ecological risk of trace elements and polycyclic aromatic hydrocarbons in sediments from a polluted urban river in central Bangladesh. Environ Nanotechnol Monit Manag 14:100318. https:// doi. org/ 10. 1016/j. enmm. 2020. 100318

Khan, R., Das, S., Kabir, S., Habib, M.A., Naher, K., Islam, M.A., Tamim, U., Rahman, A.K.M.R., Deb, A.K., Hossain, S.M., 2019. Evaluation of the elemental distribution in soil samples collected from ship-breaking areas and an adjacent island. *Journal of Environmental Chemical Engineering,* 7, <https://doi.org/10.1016/j.jece.2019.103189>.

Long, E.R., MacDonald, D.D., Severn, C.G., Hong, B.C., 2000. Classifying probabilities of acute toxicity in marine sediments with empirically derived sediment quality guidelines. Environ. Toxicol. 19, 2598–2601.

Maftei, A.E., Buzatu, A., Buzgar, N., Apopei, A.I., 2019. Spatial Distribution of Minor Elements in the Tazlău River Sediments: Source Identification and Evaluation of Ecological Risk. Int. J. Environ. Res. Public Health, 16:4664. https://doi.org/10.3390/ijerph16234664

Müller, G., 1979. Schwermetalle in den sediments des rheins-veran-derngren seitt. 1971. Umschan. 79, 778–783.

Randa, Z., Frána, J., Mizera, J., Kucera, J., Novák, J.K., Ulrych, J., Belov, A.G., Maslov, O.D., 2007. Instrumental neutron and photon activation analysis in the geochemical study of phonolitic and trachytic rocks. Geostand. Geoanalytical Res, 31, 275-283. <https://doi.org/10.1111/j.1751-908X.2007.00839.x>.

Tamim, U., Khan, R., Jolly, Y.N., Fatema, K., Das, S., Naher, K., Islam, M.A., Islam, S.M.A., Hossain, S.M., 2016. Elemental distribution of metals in urban river sediments near an industrial effluent source. Chemospher155 509e518. https://doi.org/10.1016/j.chemosphere.2016.04.099
